# Strong oral plaque microbiome signatures for dental implant diseases identified by strain-resolution metagenomics

**DOI:** 10.1038/s41522-020-00155-7

**Published:** 2020-10-30

**Authors:** Paolo Ghensi, Paolo Manghi, Moreno Zolfo, Federica Armanini, Edoardo Pasolli, Mattia Bolzan, Alberto Bertelle, Federico Dell’Acqua, Ester Dellasega, Romina Waldner, Francesco Tessarolo, Cristiano Tomasi, Nicola Segata

**Affiliations:** 1 Department CIBIO, University of Trento, Trento, Italy; 2 Private Practice, Trentino-Alto Adige, Italy; 3 Department of Industrial Engineering, University of Trento, Trento, Italy; 4 Healthcare Research and Innovation Program (IRCS-FBK-PAT), Bruno Kessler Foundation, Trento, Italy; 5 Department of Periodontology, Institute of Odontology, Sahlgrenska Academy, University of Gothenburg, Gothenburg, Sweden; 6 Present Address: PreBiomics S.r.l., Trento, Italy

**Keywords:** Plaque, Metagenomics

## Abstract

Dental implants are installed in an increasing number of patients. Mucositis and peri-implantitis are common microbial–biofilm-associated diseases affecting the tissues that surround the dental implant and are a major medical and socioeconomic burden. By metagenomic sequencing of the plaque microbiome in different peri-implant health and disease conditions (113 samples from 72 individuals), we found microbial signatures for peri-implantitis and mucositis and defined the peri-implantitis-related complex (PiRC) composed by the 7 most discriminative bacteria. The peri-implantitis microbiome is site specific as contralateral healthy sites resembled more the microbiome of healthy implants, while mucositis was specifically enriched for *Fusobacterium nucleatum* acting as a keystone colonizer. Microbiome-based machine learning showed high diagnostic and prognostic power for peri-implant diseases and strain-level profiling identified a previously uncharacterized subspecies of *F. nucleatum* to be particularly associated with disease. Altogether, we associated the plaque microbiome with peri-implant diseases and identified microbial signatures of disease severity.

## Introduction

Since the late 70s, the use of dental implants to replace missing teeth has become an increasingly common clinical practice and continuous technological innovations have now made implant therapy more reliable and accessible to the population^1–4^. Approximately 12 million implants are placed every year worldwide^5,6^, but the past 3 decades have seen the emergence of two new oral diseases: peri-implantitis, which affects both the soft and hard tissues surrounding the implant, and mucositis, which precedes peri-implantitis and involves instead only the soft tissues^7–11^. Mucositis affects >50% of the implants, while almost 20% of implants develop peri-implantitis^12–16^. About half of all inserted dental implants are thus suffering from diseases, leading in most cases to implant loosening or to the need for implant removal^17,18^ with very large clinical and socio-economical burdens and serious impairment of patients’ quality of life^19–22^.

Peri-implant diseases are clinically characterized by soft tissue inflammation (mucositis), which may lead to loss of supporting bone (peri-implantitis)^14–16^. The disease is the result of a continuum of inflammation, tissue destruction, and microbial pressure. Similarly to what is known for periodontal diseases, these factors are influenced also by host-specific immune-mediated response and genetics and are partially modulated by lifestyle and environmental factors^23^. Despite significant advances made in both periodontal microbiology and pathobiology, it is still unsettled whether the primary disease trigger is the microbial challenge or the hyperinflammatory state itself^24^. However, differently from the case of bacteria associated with periodontal diseases that have been studied for decades, the only recent emergence of peri-implant diseases limited similar investigations for mucositis and peri-implantitis. Although some known periodontitis-associated bacteria may also be connected with peri-implant diseases, different microorganisms have been suggested to be involved in these two clinically distinct conditions^25–29^. A thorough profiling of the microbiome associated with peri-implant diseases is thus an important step to undertake to then help better contextualize host response, genetics and environmental factors, and start moving toward the development of diagnostic, preventive, and therapeutic approaches.

Currently available studies investigating the link between the plaque microbiome and peri-implantitis have, however, important limitations. First, they all rely on the 16S rRNA gene sequencing approach^30^ to profile the plaque microbiome^26,31–56^. Compared to the shotgun metagenomic approach^57–59^, this cost-effective technique is unable to identify most microbes at the species level and cannot profile the overall functional potential of the microbial community. Second, the oral human microbiome^60,61^ requires large sample sizes and intra-subject microbiome controls (i.e., contralateral sites) to account for its large inter-subject variability that are not considered in the available studies^35,39,40,46,47,49,51,55,56^. A recent study sampled multiple implants from the same individual but on a small cohort (*n* = 18) of also periodontal patients^55^. Third, mucositis and peri-implantitis are very related diseases that should be considered together, but the only two works in which both conditions were sampled focused specifically on the role of smoking on the plaque microbiome^42^ or had a very small sample size and no intra-subject controls^43^. There is thus the need for a high-resolution metagenomic investigation of a large cohort including both mucositis and peri-implantitis and controlling for inter-subject variability.

In this work, we investigated the plaque microbiome associated with peri-implantitis and mucositis in a cohort of 72 patients by sequencing a total of 113 metagenomes to identify specific microbial signatures associated with peri-implant diseases. Our study design included, as controls, both healthy implants and teeth that were sampled from healthy sites from healthy individuals and from the contralateral healthy site with respect to the mucositis or peri-implantitis sites. The analysis included not only the investigation of the quantitative taxonomic composition of the plaque microbiome but we also explored the functional potential of the microbiome and the specific strains populating these plaque microbial communities.

## Results

### A metagenomic cohort to study the plaque microbiome in peri-implant diseases

In order to study the role of the oral microbiome in peri-implant diseases (mucositis and peri-implantitis), we performed shotgun metagenomic sequencing^57^ of the plaque microbiome of 113 samples from individuals with dental implants. We profiled subjects with only healthy implants (H group, *n* = 35), at least an implant with mucositis (M group, *n* = 37), and at least an implant with peri-implantitis (P group, *n* = 41) (Table 1). Submucosal and subgingival plaque samples were collected with sterile titanium Gracey curettes at the time of diagnosis by expert periodontists in six Italian dental private practices following the same standardized and validated protocol. For each patient, two distinct sites were sampled: the implant in one of the three conditions and the corresponding healthy contralateral implant. The contralateral tooth was sampled only when a contralateral implant was not available (Supplementary Data S1 and S2). The non-diseased contralateral controls were sampled to assess intra-subject variability and the level of localization of the peri-implant-specific microbiome. The inclusion of subjects in the three study groups was based on radiographic evaluation of the marginal bone level around the implant, inflammation status (bleeding on probing (BOP)), and presence of pus (suppuration (SUP)) according to the validated clinical criteria when the study was designed according to the Consensus Report on Peri-implant Diseases^8^ (see “Methods”).

**Table 1:** Demographic, anamnestic, and clinical characteristics of the sampled population.

	Healthy	Mucositis	Peri-implantitis	*p* value
No. of subjects	24	24	24	—
Age (mean (range))	62 (45–77)	63 (43–86)	62 (42–78)	0.83
Gender (M/F)	14/10	10/14	14/10	0.41
History of periodontitis (Y/N)	5/19	11/13	11/13	0.12
Smoking (Y/N)	2/22	7/17	7/17	0.13
Diabetes (*n* cases)	1	0	2	0.16
No. of implants (mean (SD))	3.6 (2.5)	3.8 (2.2)	4.1 (3.0)	0.82
No. of teeth (mean (SD))	20.1 (7.0)	17.8 (8.1)	18.4 (8.5)	0.20
Previous peri-implantitis (Y/N)	2/22	4/20	6/18	0.30
Frequency of home oral care (mean (SD))	2.1 (0.7)	2.3 (0.9)	2.0 (0.7)	0.33

Study groups significantly differed for the main known clinical parameters of disease, namely, peri-implant probing depth (PPD), BOP, SUP, and peri-implant bone loss (see “Methods”). The three clinical conditions were not confounded by other factors such as demographic, anamnestic, clinical, and implant-related characteristics (Table 1 and Supplementary Data S1 and S2). Shotgun metagenomics produced 113 quality-controlled samples for a total of ~1.1 × 10^11^ bases (Supplementary Data S3). Quantitative taxonomic profiling of the metagenomes detected a total of 288 species present in at least one sample (average 10.7, st. dev. 11.2 per sample), and an overall functional potential of 558,882 microbial gene families. Consistently with two other dental plaque metagenomic datasets^62,63^, we found that the bacterial fraction of the community quantitatively dominates over archaeal, micro-Eukaryotic microbes, and viral components (Supplementary Fig. 1), and we thus performed the analysis on the bacterial microbiome only.

### A strong microbiome signature in peri-implantitis sites

**Fig. 1:**
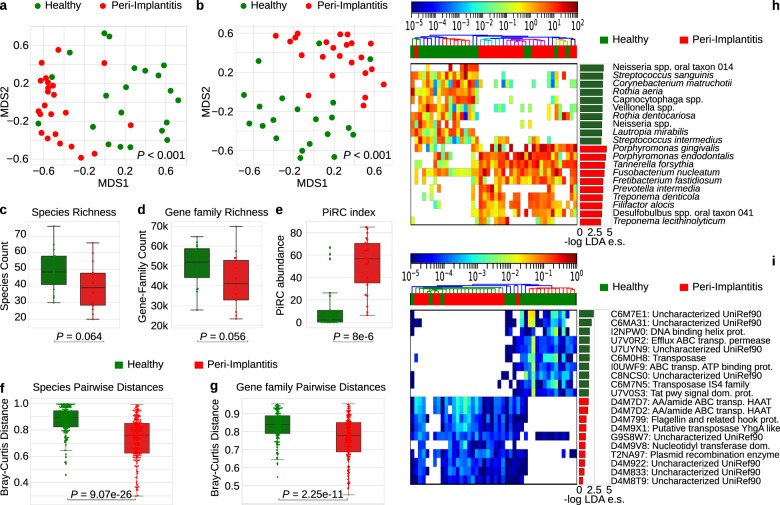
The plaque microbiome strongly differs in healthy and peri-implantitis sites. **a** Multidimensional scaling (MDS) ordination plot of healthy and peri-implantitis sites based on the Bray–Curtis distance between microbiome samples highlights a strong condition-specific clustering. *p* values were obtained by PERMANOVA. **b** Ordination plot (MDS) of healthy and peri-implantitis metagenomic samples based on the abundance of microbial UniProt90 gene families. *p* values were obtained by PERMANOVA. **c** Alpha-diversity distributions computed after rarefaction to the same sequencing depth and estimated as the richness of species and **d** as richness of UniProt90 gene families show a trend of decreased diversity in peri-implantitis sites. *p* values were obtained by the two-tailed Wilcoxon signed-rank test. **e** Distribution of PiRC index values (the total abundance species in the peri-implantitis-related complex) in healthy and peri-implantitis samples. *p* value was obtained by the two-tailed Wilcoxon signed-rank test. **f** Intra-condition beta-diversity (Bray–Curtis dissimilarity) for microbial species and **g** gene families points at a converging microbiome structure in diseased peri-implantitis sites. *p* values were obtained by two-tailed Wilcoxon signed-rank test. **h** Relative abundances (log scale) and effect sizes (LDA score from LEfSe) of the 10 microbial species and **i** gene families most strongly associated with either the healthy sites or the sites with peri-implantitis (top 10 species/gene families effect sizes per class). All the comparisons in this figure are performed using the sample from the main site in each subject without considering the contralateral samples.

Analysis of the quantitative taxonomic composition of the plaque microbiome via MetaPhlAn2^64^ (see “Methods”), highlighted a clear distinction between the plaque from sites with peri-implantitis and from healthy implants in healthy individuals (Fig. 1a, only the main site considered, permutational multivariate analysis of variance (PERMANOVA) *p* value < 0.001). The overall functional potential of the microbial communities of the plaque assessed using HUMAnN2^65^ (see “Methods”) was also clearly distinctive for the two conditions (PERMANOVA *p* value < 0.001) at the level of abundance of single-gene families (Fig. 1b) as well as whole-microbial pathways (Supplementary Fig. 2a). The two conditions showed distinct alpha-diversity profiles of the biofilm, with the richness of species, genes, and pathways substantially reduced in peri-implantitis compared to controls (Fig. 1c, d and Supplementary Fig. 2b), although inter-subject variability of alpha diversity prevents strong statistical support (Wilcoxon signed-rank *p* value = 0.064 and 0.056 for species and gene family richness, respectively). This trend points at a plaque microbiome in peri-implantitis composed by fewer dominant species that are encoding a smaller number of functions. The healthy plaque was not only richer but also more variable among individuals (Fig. 1f, g), while diseased implants were characterized by microbiome structures converging toward a more well-defined composition (Wilcoxon signed-rank *p* value = 9.07e−26, *p* value = 2.25e−11 on beta-diversity groups when considering taxonomic and functional potential profiles, respectively). At the whole-microbiome level, peri-implantitis is thus associated with a relatively low diversity microbiome with a well-defined and inter-patient conserved set of bacterial taxa.

The taxonomic composition of the peri-implant microbiome was characterized by 71 species that were differentially abundant (Wilcoxon test *p* value < 0.05) between the disease and healthy state, with 54 of them passing multiple hypothesis testing correction (false discovery rate (FDR) *q* < 0.1, Supplementary Data S4 and S5). Among them, 12 species were enriched in peri-implantitis and accordingly had very high prevalence in diseased sites, with 10 of these 12 species at >73% prevalence in peri-implantitis ranging from the 100% of *Tannerella forsythia* to 52% of *Treponema lecithinolyticum*. The same species had lower but not negligible prevalence in the control sites with *T. forsythia*, *Treponema socranskii*, and *Fusobacterium nucleatum* exceeding 50% prevalence in healthy conditions (Supplementary Data S4). The highest effect size between peri-implantitis and controls was detected for *Porphyromonas gingivalis* and *Porphyromonas endodontalis* (Fig. 1h), but all the 12 peri-implantitis-associated species are substantially more abundant in the disease compared to the healthy state with mean enrichments above 10× for *Treponema maltophilum*, *Fretibacterium fastidiosum*, *Pseudoramibacter alactolyticus*, and *T. lecithinolyticum*. Conversely, 16 species significantly associated with the healthy implants and with >40% prevalence in controls were never detected in the peri-implantitis group, suggesting that several different taxa including multiple *Capnocytophaga* (*C. gingivalis*, *C. granulosa*, *C. ochracea*) and *Selenomonas* (*S. noxia*, *S. artemidis*) species characterize the complex and diverse healthy plaque. Most healthy-associated species belonged to the genera *Actinomyces* (7 species), *Capnocytophaga* (4 species), *Neisseria* (4 species), *Rothia* (3 species), and *Streptococcus* (5 species) that have no species associated with peri-implantitis.

The different taxonomic composition in the two disease states were reflected at the level of the functional repertoire of the species with many gene families differentially abundant (22,982 FDR-corrected gene families) and again very clearly differentiating the two conditions (Fig. 1i and Supplementary Data S6 and S7). Overall, statistical significant biomarkers (Fig. 1h, i and Supplementary Fig. 2d) of either peri-implantitis or healthy implants constituted a large fraction of the plaque microbiome (average 63.6% s.d. 23.9% relative abundance when considering the 54 FDR-corrected species), indicating profound differences in bacterial populations associated with peri-implantitis and healthy conditions.

### The “peri-implantitis-related complex (PiRC)” comprises the seven most disease-associated species

Several species that are strongly associated with peri-implantitis are in the set of bacteria recently linked to periodontal disease^66^. Considering the ten most strongly peri-implantitis-associated species (Fig. 1h), the three most common periodontitis-associated bacteria belonging to Socransky’s Red Complex are ranked first (*P. gingivalis*), third (*T. forsythia*), and seventh (*Treponema denticola*) based on their effect size. This highlighted that *P. endodontalis* (second in the ranking) and *F. fastidiosum* (fifth) should be considered in the same class of key species for peri-implantitis. The other three bacteria in the ten strongest peri-implantitis-associated species (*Filifactor alocis*, *Desulfobulbus* spp. oral taxon 041, and *T. lecithinolyticum*) are also newly associated with the disease, but because they show a smaller effect size compared to the red complex triad, we considered them comparatively less discriminative. We thus propose to define the PiRC of species strongly characterizing peri-implantitis sites as the set of microbes composed by the red complex triad, by the two newly associated *P. endodontalis* and *F. fastidiosum* species, and by the *Prevotella intermedia* and *F. nucleatum* species found associated with peri-implantitis at higher effect size than *T. denticola*, the third bacterium of the red complex triad. The PiRC index defined as the cumulative abundance of the species in the PiRC complex is highly discriminative for the two conditions (Fig. 1e) and may be a relevant summary index for clinical evaluation of the peri-implant microbiome.

### The peri-implantitis microbiome signature is site specific

**Fig. 2:**
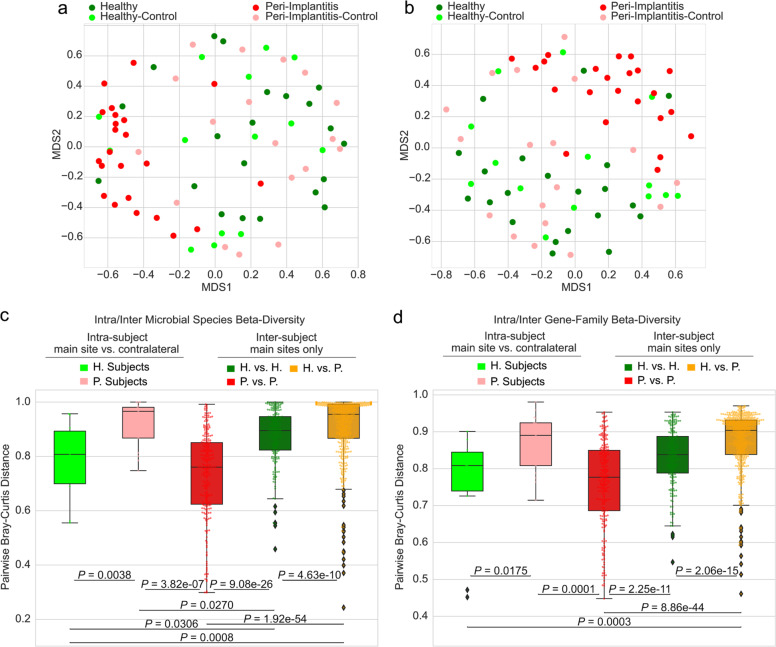
The microbiome in peri-implantitis patients is site specific and peri-implantitis sites are microbially consistent across individuals. **a** Ordination plot (MDS) of peri-implantitis and healthy metagenomic samples (main and contralateral) based on the Bray–Curtis distances highlights the clustering of healthy contralateral samples in peri-implantitis with the samples from healthy individuals. **b** Ordination analysis as per **a** obtained using microbial gene family abundances instead of taxonomic profiles. **c** Beta-diversities estimated with the Bray–Curtis dissimilarity metric for intra- and inter-condition comparisons in the diseased and healthy conditions estimated using the taxonomic microbial composition and **d** the functional microbial gene family composition. *p* values were obtained by two-tailed Wilcoxon signed-rank test and reported if considered significant (<0.05). Estimations of the intra-subject beta-diversity are performed using the main site and the contralateral samples.

Peri-implantitis may affect independently different dental implants inside the same oral cavity, but whether the associated microbiome is also site specific is currently unclear and debated^34,35,38,67–71^. We thus considered samples from healthy contralateral implants (*n* = 10) and teeth (*n* = 22) in the same volunteers who were sampled from healthy and diseased implants (Fig. 2a). Contralateral implants and teeth were considered together, because we observed no significant difference in the microbiome associated with main healthy sites and contralateral healthy sites both taxonomically and functionally (Supplementary Fig. 4a–c, PERMANOVA *p* value = 0.59 when considering species-level abundances, 0.57 when looking at gene families). We found that the microbiome samples of contralateral healthy sites clustered together with the healthy samples regardless of the disease status of the main sites. Indeed, while as expected healthy main sites and their healthy contralateral sites in subjects without peri-implantitis do not have different microbiomes (PERMANOVA *p* value = 0.58, no differential species after FDR correction), the healthy contralateral sites of main peri-implantitis sites do not differ from main healthy sites in volunteers without peri-implantitis (PERMANOVA *p* value = 0.55 with no differential species after FDR correction) nor from the healthy contralateral sites of healthy sites (PERMANOVA *p* value = 0.53, no FDR-corrected differential species). Peri-implant sites have instead clearly distinct microbiomes from their healthy contralateral sites (PERMANOVA *p* value < 0.001, 15 FDR-corrected differential species), from main sites of healthy subjects (PERMANOVA *p* value < 0.001, 11 FDR-corrected differential species), and from contralateral sites of healthy subjects (PERMANOVA *p* value = 0.001, 6 FDR-corrected differential species). These findings were confirmed by the analysis of the functional potential of the microbiome (Fig. 2b and Supplementary Fig. 3a). The peri-implantitis microbiome is thus strongly site-specific as the microbial composition of the plaque of healthy implants is indistinguishable in subjects with and without peri-implantitis in other implants.

Site specificity of the peri-implantitis microbiome is further confirmed by beta-diversity analysis (Fig. 2c). The Bray–Curtis microbiome distances between main and contralateral sites were significantly lower when both sides are healthy reflecting the peri-implantitis shift in microbiome composition. This holds true also when considering the abundance of gene families (Fig. 2d) and of whole-microbial pathways (Supplementary Fig. 3b). Inter-subject taxonomic and functional microbiome distances (Fig. 2c, d) also confirm that peri-implantitis-associated microbiomes tend to converge toward a specific configuration, whereas the plaque microbiome of healthy sites is more diverse and different between subjects. It is also of note that, for healthy sites (main or contralateral), the plaque microbiome of implants and teeth is not distinguishable (Supplementary Fig. 4a–c), which is further reinforcing the site specificity of the detected peri-implantitis microbiome signature.

### *F. nucleatum* as a key species in the intermediate microbial signature of mucositis

**Fig. 3:**
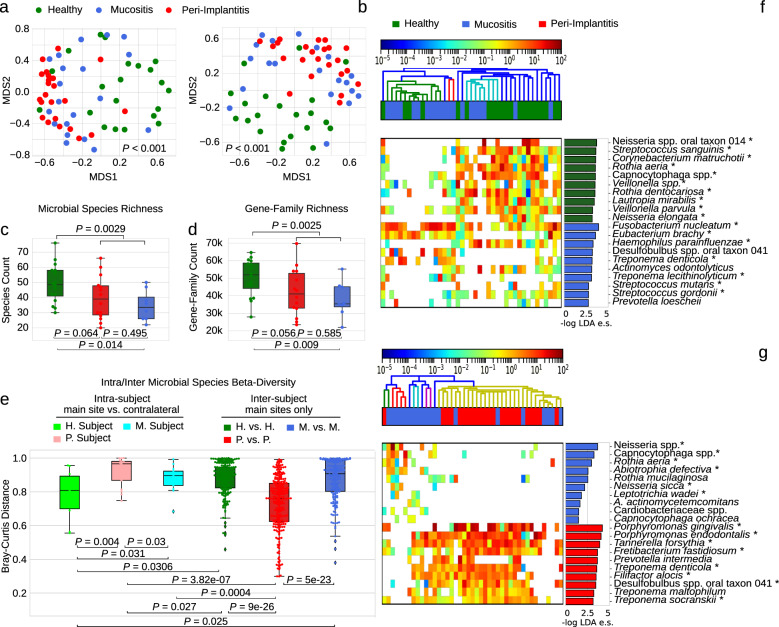
Mucositis shows an intermediate microbial signature between peri-implantitis and healthy sites. **a** Ordination plot (MDS) of healthy, mucositis, and peri-implantitis samples based on taxonomic abundance profiles and **b** microbial gene family profiles. **c** Alpha-diversity distributions estimated as species richness and **d** richness of UniProt90 gene families. **e** Beta-diversity distributions estimated with the Bray–Curtis dissimilarity metric for intra- and inter-condition comparisons in the three conditions. Estimations of the intra-subject beta-diversity are performed using the main site and the contralateral samples. **f** Relative abundances (log scale) and effect sizes (LDA score from LEfSe) of the ten microbial species most strongly associated with mucositis in comparison with healthy sites and **g** peri-implantitis sites (top ten species/gene family effect sizes per class).

Mucositis is an inflammatory intermediate condition that is a prerequisite for peri-implantitis^11,14,15^. To characterize its associated microbiome, we sampled the plaque of 20 mucositis-affected implants (and their non-diseased contralateral sites) and assessed its structure as compared to healthy individuals and peri-implantitis patients. Quantitative microbiome profiles (Supplementary Figs. 5d, e and 6) based on taxonomic (Fig. 3a) and functional potential profiles (Fig. 3b and Supplementary Fig. 5a) did not highlight a clear discrete sample clustering distinctive for mucositis. Despite its intermediate position, mucositis still, however, significantly differs in its associated microbiome compared to both the peri-implantitis group (PERMANOVA *p* value = 0.008) and healthy implant control group (PERMANOVA *p* value = 0.001) irrespective of potential confounding factors including sampling procedures (Supplementary Fig. 6). For alpha diversity, the mucositis-associated microbiome resembled that of the peri-implantitis group with species, gene families, and pathway richness (Fig. 3c, d and Supplementary Fig. 5b) substantially depleted compared to the healthy plaque microbiome. This is in line with the hypothesis of a biofilm composed by fewer dominant species in disease and a consequent small number of inferred gene functions. On inter-subject microbiome divergence, mucositis is again in between healthy and peri-implantitis sites both from the taxonomic (Fig. 3e) and functional potential viewpoint (Supplementary Figs. 5c and 7a), suggesting that the mucositis-associated microbiome has a converging composition and structure that is less marked than in full-disease states.

*F. nucleatum* and *T. denticola* are the only species within the ten most mucositis/healthy discriminative species and also included in the seven-species PiRC defined above (Fig. 3f). Conversely, the four of the five species that better distinguish peri-implantitis with respect to mucositis all belong to the PiRC, with only *F. nucleatum* not present in the list (Fig. 3g). These observations suggest that *F. nucleatum* is the first species that increases in abundance inside the plaque microbiome along the mucositis–peri-implantitis axis and is then followed by more strongly peri-implantitis characterizing species at later stages of the disease. The microbiome signatures of mucositis and peri-implantitis are thus distinct and understanding the transition from the first to the second could provide important therapeutic targets.

### PiRC bacteria are responsible for the microbial functions enriched in peri-implantitis

*F. nucleatum* appeared as a key actor in the mucositis-associated microbiome not only for its increased presence but also for its contribution to the overrepresented microbial pathways in this condition (Supplementary Fig. 8). This is highlighted in the HUMAnN2-based analysis of the ten top-ranking pathways in peri-implant conditions (Figs. 1h and 3g) where *F. nucleatum* and *P. endodontalis* are responsible for most of the differential microbial pathways. The relative contribution to the peri-implantitis-enriched microbial pathways is similarly dominated by the bacteria in the PiRC that are the highest contributors in 265 of the total 414 subject–pathway associations (64%). In particular, *P. endodontalis* is the greater contributor, being the main responsible for 95 associations (36%), followed by *T. forsythia* (85 associations, 32%).

### Microbiome-based classifiers can accurately predict clinical peri-implant conditions and disease progression

**Fig. 4:**
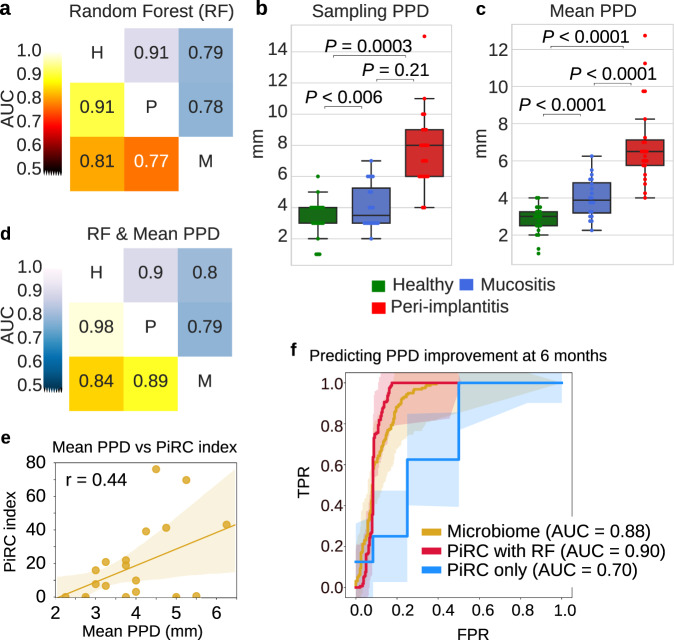
Diagnostic and prognostic metagenomic machine-learning signatures for mucositis and peri-implantitis. **a** AUC prediction matrix between pairs of conditions (H healthy, M mucositis, P peri-implantitis) achieved by the random forest machine-learning classifier using taxonomic species-level features (bottom left triangular matrix in black–red–yellow colormap) and functional gene family features (upper right triangular matrix in blue colormap). Classifiers are applied in 10-fold cross-validation repeated 20 times. **b** Distribution of PPD values at the sampling site (“sampling PPD”) and **c** mean PPD for the three conditions considered in this study. **d** The same prediction matrix of **a** but using a classifier trained both on microbiome features (species or gene family abundance) and mean PPD values. Only samples from the main peri-implant sites are used in these analyses without considering the contralateral sites. **e** Correlation between mean PPD at baseline and peri-implantitis-related complex (PiRC) index for the mucositis samples. Pearson’s correlation coefficient is reported. **f** Receiver operating characteristic (ROC) curves and AUC values for the cross-validation performance of the microbiome-based RF classifier in predicting improvements in mean PPD values. The three models consider (i) all species, (ii) the species in the PiRC, and (iii) the PiRC index as input features.

We then assessed the potential diagnostic and prognostic potential of the plaque microbiome for mucositis and peri-implantitis. Using a validated machine-learning approach based on the random forest classifier^72^ applied in cross-validation (see “Methods”)^73^, we found that peri-implantitis can be predicted at high accuracy, with an area under the receiver operating characteristic curve (AUC) of 0.91 (Fig. 4a). Mucositis was also clearly associated with its microbiome but with comparably less precision and recall for the task of predicting mucositis versus healthy conditions (AUC = 0.81) and mucositis versus peri-implantitis (AUC = 0.77). Similar classification performances were obtained when using gene families for training the classifier (Fig. 4a), suggesting that the species that are discriminative for the disease conditions possess distinctive sets of genes.

An important parameter that is collected when assessing and monitoring the clinical condition of an implant is the PPD at multiple sites around the implant. Such parameter is clearly distinctive for peri-implantitis but weaker for mucositis (Fig. 4b, c). Mean PPD alone can reach an AUC of 0.95 in predicting peri-implantitis versus healthy sites (Supplementary Fig. 9a), but for mucositis it reached worse performances than the microbiome-based classifier with AUC 0.73 (Supplementary Fig. 9a) versus AUC 0.81 (Fig. 4a). When combining the microbiome taxonomic features with the PPD values, the classifier outperformed the microbiome- and PPD-based classifiers with very high AUCs for all pairs of conditions (Fig. 4d). With an AUC 0.98 for the combined classifier, microbiome and clinical parameters thus provide complementary lines of evidence for predicting the implant disease status.

Because mucositis is the most critical clinical condition for its risk of evolution in peri-implantitis, we then tested whether the microbiome associated with mucositis can be a good proxy for the severity of the condition. First, for the mucositis samples we analyzed (that were not used in the definition of the PiRC), we found that the PiRC index is correlated with mean PPDs for the mucositis samples (Fig. 4e, Pearson’s *R* = 0.44, *p* value = 0.049). Second, using PPD values at 6 months as a proxy for improvement or worsening of the clinical condition, we found that the machine-learning classifier trained in cross-validation on the mucositis microbiome was predictive for positive or negative PPD variation (AUC 0.88, Fig. 4f). This was confirmed by the use in the classifier of the 7 species in the PiRC alone (AUC 0.90, Fig. 4f) as well as just using the PiRC index (AUC 0.70, Fig. 4f). This demonstrates that the plaque microbiome associated with mucositis is predictive for the variation in clinical parameters and can potentially be used as a tool for testing the severity of the clinical condition.

### Specific *F. nucleatum* subspecies are associated with mucositis and peri-implantitis

**Fig. 5:**
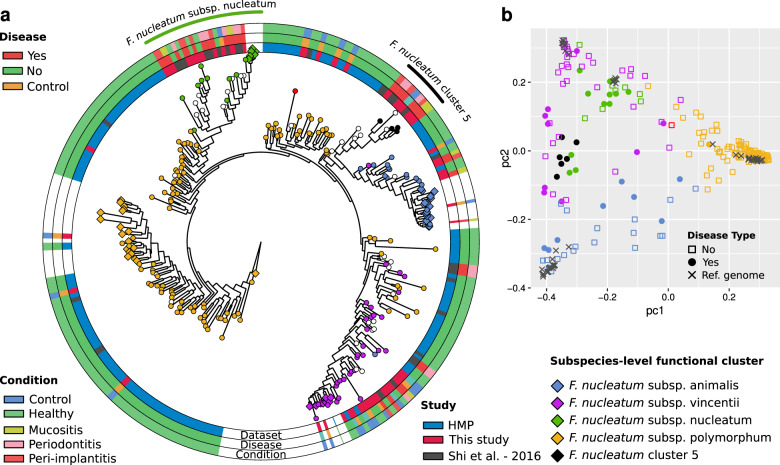
Strain-level characterization of *Fusobacterium nucleatum* from metagenomes of the healthy, mucositis, peri-implantitis, and periodontitis plaque. **a** StrainPhlAn-based phylogenetic tree computed on the MetaPhlAn2 markers for *F. nucleatum*. Each node represents a single *F. nucleatum* strain reconstructed from a sample. Clinical metadata are reported in the three outer rings. Contralateral controls of healthy patients are labeled as “healthy.” Nodes are colored by genomic-content cluster (“Methods” and Supplementary Fig. 10). White nodes reflect samples that could not be detected and typed by PanPhlan and that were not clustered. Reference genomes are indicated by diamond leaf markers. The five colors of the markers refer to the five identified subtypes, including the newly identified *F. nucleatum* Cluster 5 subspecies (see “Methods”). Subspecies significantly associated with peri-implant diseases are marked externally to the diagram. **b** Clustering and principal coordinates analysis of the PanPhlAn-detected *F. nucleatum* strains. The analysis is performed on the gene presence–absence profiles of *F. nucleatum* pangenome. Points are colored by genomic-content cluster (“Methods” and Supplementary Fig. 10).

*F. nucleatum* is a key bacterium in the plaque microbiome associated with periodontal diseases^74,75^, and we showed here also that it is strongly associated with peri-implant diseases and especially with mucositis. Given the diversity of the strains present in this species^76^, we further investigated the subspecies structure of *F. nucleatum* in relation to peri-implantitis and mucositis (see “Methods”). By using a pangenome-based strain profiling tool^77^, we characterized the dominant *F. nucleatum* strains in the 113 samples considered in this study as well as in 135 additional plaque samples from an American healthy metagenomic cohort^63^ and 48 plaque samples from patients with periodontitis^62^. We recovered a total of 208 *F. nucleatum* strain profiles that were clustered into five distinct subspecies (Supplementary Fig. 9). These clusters correctly recapitulated the known subspecies of *F. nucleatum*^78^, which are subspp. nucleatum, polymorphum, Vincentii, and animalis (Supplementary Fig. 10), but also identified the presence of one additional yet-to-be-named subspecies without taxonomic annotations we provisionally named *F. nucleatum* Cluster 5. We then profiled the strains also using the genetic variability of their core genome^79^ which confirmed that *F. nucleatum* subspecies were phylogenetically well separated, with reference genomes monophyletically placed, and in agreement with the genomic-content clustering. Metagenomes harboring strains of the “animalis” and “nucleatum” subspecies were mostly comprised of monophyletic subtrees, while samples with the “polymorphum” cluster were more diverse (Fig. 5a).

We then tested the association of specific subspecies with the clinical conditions. Strains belonging to *F. nucleatum* subsp. nucleatum were significantly more associated with peri-implantitis or mucositis sites (*p* value = 2.27e-4, two-tailed Fisher’s exact test) than healthy sites. Conversely, *F. nucleatum* subsp. polymorphum was enriched in healthy subjects and control sites. Interestingly, the newly identified and unexplored *F. nucleatum* Cluster 5 was significantly associated with peri-implantitis and mucositis samples, when compared to healthy subjects and controls (*p* value = 3.05e-5, two-tailed Fisher’s exact test). Three additional periodontitis samples harbored a member of *F. nucleatum* Cluster 5, and the association of this subspecies with disease status held true even when including samples with periodontitis (*p* value = 2.61e−5). Taken together, these results suggest that only a subset of *F. nucleatum* strains including a newly identified subspecies without cultivated representatives is associated with peri-implant diseases and identifying *Fusobacterium* subspecies in the clinic may have prognostic relevance.

## Discussion

In this work, we profiled the microbial composition of the plaque biofilm in implant diseases overcoming in resolution (shotgun metagenomics versus 16S rRNA sequencing), sample size (113 metagenomes), and inter-individual microbiome control (contralateral sites) the existing investigations. We found strong taxonomic and functional biomarkers for disease and highlighted how peri-implantitis converges toward a low-diversity well-defined pathogenic set of taxa. Importantly, the link that we characterized between peri-implant disease state and the plaque microbiome is substantially stronger than what was previously observed for other oral conditions that can have a role in peri-implantitis such as smoking^80^ and diabetes^81,82^. The seven bacterial species we included in the PiRC are potential appealing targets for diagnostic and prognostic assessment of the plaque microbiome and the basis for more targeted mechanistic investigation of the host–microbiome interaction network in implant-associated diseases.

Our data also suggest that *F. nucleatum* is a particularly relevant member of the PiRC because it appears at an initial stage in the inflammation (mucositis). This species is indeed overly abundant before the onset of peri-implantitis and precedes the stronger microbial shift involving many additional taxa. *F. nucleatum* is a particularly diverse species, and of its known subspecies, only *F. nucleatum* subsp. nucleatum is connected with disease states. A new subspecies provisionally named *F. nucleatum* Cluster 5 is even more associated with peri-implantitis and mucositis and should be the basis for further characterization of this clade.

Mucositis is perhaps the more actionable disease state, and although even larger sample sizes are needed, its intermediary microbiome configuration that appears to be in between the healthy plaque and the peri-implantitis plaque is of primary relevance to better understand the molecular cascade in peri-implant diseases. We indeed found that the improvement of clinical parameters associated with mucositis (such as probing depth) that we surveyed longitudinally can be predicted starting from the microbiome structure at diagnosis. Specific microbiome-based therapeutic choices are currently not available, but the presence and abundance of *F. nucleatum* and other taxa of the PiRC might be associated with more or less favorable outcomes. More work is thus needed to correlate the structure of the mucositis-associated microbiome with the progress of the disease state.

The high-resolution characterization of the plaque microbiome in peri-implant diseases is the first step toward a more comprehensive understanding of the role of the microbiome in peri-implant diseases. Although the inter-subject diversity of the peri-implantitis microbiome is relatively low, the predominance of different taxa in the PiRC might be useful for disease stratification. Further stratifying the three main disease classes using larger sample sizes for a series of covariates that are relevant for oral health such as diabetes, smoking, alcohol, and previous history of periodontitis will also be important to refine the microbiome-based models. In addition, the strong signature and the site specificity of the microbiome in peri-implantitis suggest personalized medicine perspectives based on the local use of antibiotics or antiseptics. Local rather than systemic administration would allow to target PiRC taxa minimizing the impact on the entire oral ecology. Further studies are thus needed to define microbiome-dependent disease subtypes and test the efficacy of therapeutic strategies on such subtype stratification.

## Methods

### Subject recruitment

This study was approved by the ethics committee of the University of Trento (no. 2015-024) and was conducted in accordance with the guidelines of the World Medical Association Declaration of Helsinki. Male and female patients having dental implants and regular maintenance of their dental implants were recruited from six different private practices in the Province of Trentino (Italy) for this study. The study protocol was explained to each subject, and a signed informed consent was obtained. Inclusion criteria involved good general health as evidenced by the medical history, being at least 18 years of age, not fewer than 8 teeth, at least one functioning oral implant restored with crowns or prostheses for at least 1 year, willingness to participate in the study. Exclusion criteria included pregnancy or lactation, human immunodeficiency virus, use of immunosuppressant medications, bisphosphonates, or steroids, use of chlorhexidine mouthwash or gel during the previous 2 weeks, oral prophylactic procedures within the preceding 3 months, intake of systemic antibiotics or probiotics within the past 6 months.

Patients were identified and selected in the different private practices and were included in one of the following study groups according to the state of health of their dental implants: (a) healthy (H, patients with at least one healthy implant and no implants with mucositis or peri-implantitis), (b) mucositis (M, patients with at least one implant with mucositis and no implants with peri-implantitis), and (c) peri-implantitis (P, patients with at least one implant with peri-implantitis). The selection and inclusion of patients in one of the groups was based on radiographic evaluation of the marginal bone level, clinical signs of inflammation, and/or presence of SUP according to the criteria delineated by the Consensus Report on Peri-implant Diseases^8^. In detail, peri-implant health was diagnosed when the implant was surrounded by healthy soft tissue as determined by the absence of BOP or SUP and visible/detectable radiographic bone loss. Implants with only clinical signs of inflammation in one or more sites (redness, swelling, bleeding, SUP) and absence of radiographic bone loss following functional loading were classified as peri-implant mucositis, whereas implants with the presence in one or more sites of both clinical inflammation and radiographic evidence of >2 mm bone loss since the prosthesis installation (i.e., at least 1 year after loading) were diagnosed as peri-implantitis.

### Data collection and clinical examination

Six experienced dentists examined all patients. Before commencing the study, meetings were organized in order to instruct all dentists on a common protocol for the examination, collection, and measurement procedures. Plaque samples were collected by a limited number of volunteers (one for each study) in order to minimize the potential biases in the sampling methodology across the different examiners. Follow-up meetings were organized every 3 months to ensure consistency of the sampling, and statistical tests on the results (see Supplementary Fig. 6) were applied to verify the absence of strong dentist-specific batch effects.

The demographic parameters of gender and age and a comprehensive medical and dental history were recorded, followed by a full-mouth periodontal and implant clinical examination and, if necessary, a site-specific radiographic examination. Medical history comprised information about smoking habit, diabetes, autoimmune diseases or other systemic diseases, alcohol consumption, and medications taken. Dental history comprised information about current and past periodontal status, number of remaining teeth, number of implants, previous peri-implantitis, frequency of home oral care, hours since last toothbrush, and chlorhexidine usage. Clinical parameters included implant or tooth, site of sampling, diagnosis of implant age (time from installation), implant system used and nature of reconstruction (single implant, fixed or removable), type of implant retention (screw, cement, conometric), radiographic peri-implant bone loss, width of the keratinized mucosa, as well as PPD, plaque index (PI), BOP, and SUP. The latter four parameters were measured in each patient at the buccal, mesial, lingual, and distal sites of the experimental implant (healthy/mucositis/peri-implantitis) and of a healthy contralateral implant (if present) or tooth. PI, BOP, and SUP were recorded on a binary scale (presence/absence) for each surface and PPD was measured to the nearest millimeter on the scale. In case of mucositis and peri-implantitis, any eventual subsequent therapy was noted. All patients were anonymized in the clinic assigning a unique patient ID to all subjects. All downstream analyses were performed using the anonymous IDs and anonymized metadata. The mapping between patients and IDs were stored and kept uniquely at the clinic with only the responsible of each center allowed to have access to it.

### Cohort and patient’s clinical characteristics

In total, we enrolled for this study 80 patients (H: 28, M: 28, P: 24; 42 males, 38 females; mean age 60.29 ± 10.01 years) contributing with one implant per patient (two if the contralateral healthy site was an implant). Eight patients, four in the health group and four in the mucositis group, were excluded due to failure in DNA extraction or library preparation for sequencing.

Study groups comprising subjects with a healthy implant (H, *N* = 24), an implant with mucositis (M, *N* = 24) and an implant with peri-implantitis (P, *N* = 24) were compared for demographic, anamnestic, and clinical characteristics (Table 1). The analysis of the sampled population showed that no differences were apparent between these three groups for age (ANOVA *p* value = 0.83), gender (Pearson Chi-square *p* value = 0.41), history of periodontitis (Pearson Chi-square *p* value = 0.12), smoking habit (Pearson Chi-square *p* value = 0.13), diabetes (Pearson Chi-square *p* value = 0.16), number of functioning implants (*p* value = 0.82), number of residual teeth (*p* value = 0.20), previous peri-implantitis (Pearson Chi-square *p* value = 0.30), and frequency of home oral care (*p* value = 0.33). Extensive clinical data were registered both for experimental implants and contralateral implants or teeth (Supplementary Data S1 and S2). When clinical parameters for the experimental implants were considered at the implant level, a significant difference was observed for PPD (ANOVA *p* value < 0.001), BOP (Pearson Chi-square *p* value < 0.001), SUP (Pearson Chi-square *p* value < 0.001), and bone loss (ANOVA *p* value < 0.001), all these parameters significantly higher in the peri-implantitis than in the peri-implant health group. PI was instead non-significant (Pearson Chi square *p* value = 0.20) among groups (Supplementary Data S1).

### Sample collection, DNA extraction, and Illumina shotgun sequencing

Microbiome samples were collected with a non-invasive procedure adopting a sampling protocol based on the one validated and adopted by the Human Microbiome Project (HMP) consortium^63^. Samples were collected with technical replicates (two per site) from a single implant and from the healthy contralateral/implant/tooth (if present, a healthy implant was preferred as contralateral healthy site) for each patient in each study group. If multiple implants with the same tested condition were present in a patient, one single implant was randomly selected for sampling. The selected sites were isolated using cotton rolls to prevent contamination with saliva and gently dried with an air syringe, and supramucosal and supragingival plaque was removed using sterile cotton pellets. Submucosal and subgingival plaque samples were taken from the deepest probing site at each selected implant and tooth with individual sterile titanium Gracey curettes. Technical replicates were sampled only after the interruption of any eventual bleeding in order to avoid contamination of the microbiological sample. Curettes were preferred to sterile paper points according to HMP^63^ and to avoid potential contaminating bacterial DNA associated with paper points^83^. After the collection, samples were immediately placed in separate Eppendorf 1.5-mL microcentrifuge tubes (Eppendorf, Hamburg, Germany) containing sterile SCF-1 buffer solution (50 mM Tris-HCl, pH 7.5; 1 mM EDTA, pH 8.0; 0.5% Tween-20)^84^ and frozen at −80 °C for later analysis. Total genomic DNA was isolated using the QIAamp DNA Mini Kit (Qiagen, Hilden, Germany): an additional enzymatic disruption step for complete lysis of Gram-positive and Gram-negative species was performed, following the manufacturer’s protocol. Isolated DNA was stored at **−**20 °C. Laboratory control extractions were also performed on prepared sample buffer to ascertain any potential contaminants. Each metagenome was first quantified, and when there was sufficient material (>1 ng), libraries were prepared using the Nextera-XT DNA Kit (Illumina Inc., San Diego, CA, USA) using the manufacturer’s protocol. Technical replicates were used only for the cases in which the first sampling did not yield enough DNA and the DNA extraction of the second replicate was added to the first replicate. Libraries were sequenced (2 × 100 bp reads) on the Illumina HiSeq-2000 platform. Shotgun metagenomics produced initially a total of 140 samples that were reduced due to insufficient non-human DNA depth to 113 quality-controlled samples for a total of 111 Gb and with at least 10 Mb of non-human reads.

### Sequence preprocessing and taxonomic and functional potential profiling

The generated raw metagenomes were processed with FastqMcf^85^ by trimming positions with quality <15, removing low-quality reads (mean quality <25), and discarding reads shorter than 90 nt. Human and bacteriophage phiX174 DNA (Illumina spike-in) was then removed using BowTie2^86^ by mapping the reads against the corresponding reference genomes. We used MetaPhlAn 2^64,87^ with default parameter settings for the taxonomic characterization of the sampled microbial community. HUMAnN2^88^ was used to generate normalized pathway relative abundances and gene family relative abundance tables according to the metagenome gene contents. The fold change of the contributions of the PiRC Bacteria over the non-PiRC bacteria in the peri-implantitis-enriched pathways have been computed based on the HUMANn2 output.

### Statistical analysis

We performed biomarker discovery using LefSe^89^ on MetaPhlAn2 taxonomic abundance profiles and on HUMAnN2 pathway relative abundances and gene family relative abundance profiles. Alpha-diversity, beta-diversity, and multi-dimensional scaling (MDS) analysis have been performed via custom python scripts based on Scipy, version 1.2.1, and Scikit-learn, version 0.20.3^72,90^. PERMANOVA was performed using the Scikit-bio python library, ver. 0.2.3, and 1000 permutations. We used the FDR correction adopting the Benjamini–Hochberg approach implemented in the python library Statsmodel, ver. 0.9.0.

For the computation of microbiome richness we subsampled the reads to one million reads per sample. Beta-diversity was computed using the Bray–Curtis dissimilarity index after log-transformation of the raw relative abundances to minimize the effect of compositionality. The two-tailed Wilcoxon rank-sum test was used for the comparison unless otherwise stated. Metric MDS implementation, which adopts the Scaling by MAjorizing a COmplicated Function (SMACOF) algorithm to minimize the stress function, was used; the number of runs for the SMACOF was set to 4, the maximum number of iterations for each run to 5000, and the relative tolerance with respect to stress at which to declare convergence was set to 1e−09. Linear regression of the mean PPD versus the PiRC was computed and plotted together with 95% confidence intervals (Seaborn python library, ver. 0.9.0).

### Machine-learning analysis

We used a machine-learning framework similar to the one we employed in ref. ^73^, applying MetAML^72^, which is based on the Scikit-Learn implementation (ver. 0.20.3) of random forest^91^. We used random forest as this method achieved the highest consistency and lowest overfitting on metagenomic datasets over a large set of learning problems in microbiome research as reported elsewhere^72^ and demonstrated also for the analysis of the link between the gut microbiome and colorectal cancer^73^. We applied this framework on MetaPhlAn2 species-level abundance profiles, HUMANn2 gene family relative abundances, PPD at the sampling site, mean PPD across the four sites of each implant, and on combinations of these feature sets. The random forest classifier uses 1000 estimators, a maximum number of features to consider when looking for the best split of 30% of the feature set for MetaPhlAn2 and of the square root of the size of the feature set for HUMANn2. The minimum number of samples in each leaf was set to 5. The other hyperparameters were left as the default in Scikit-Learn (ver. 0.20.3). For each run, we trained and tested the model in a 20 times repeated 10-fold stratified cross-validations, evaluating the resulting predictions through the mean AUC. Models considering mucositis samples only have been trained and tested in fivefold cross-validation due to sample size constraints, and the minimum sample per leaf was consistently decreased to 1. In this case, a sample has been considered in the negative class (control), if the total millimeters of PDD surrounding the implant were decreased at 6 months after the baseline. A sample was considered positive if the total PPD millimeters were higher or equal to the baseline at 6 months.

### Strain-level analysis of *F. nucleatum*

Strain-level profiling was performed by running StrainPhlAn^79^ and PanPhlAn^77^ on the raw reads of the 113 samples considered in this study, plus 183 additional gingival plaque metagenomes from healthy and periodontitis subjects retrieved from publicly available datasets. Additionally, 56 reference genomes of *F. nucleatum* were retrieved from the NCBI Assembly database^92^ and incorporated in the strain-level analysis. For the strain-level profiling through StrainPhlAn, reads were preprocessed to remove short sequences (i.e. length <90 nucleotides) and then mapped against the MetaPhlAn2 markers with Bowtie2 v. 2.3.4^86^. StrainPhlAn was applied with the following parameters: —marker_in_clade=0.2, —sample_in_marker=0.2, —N_in_marker=0.8 and —gap_in_sample=0.8. In total, *F. nucleatum* was detected in 208 out of 296 samples. The phylogenetic maximum likelihood tree was generated on the multiple sequence alignment using Muscle v3.8.425^93^ and RAxML v.8.1.15^94^ with the GTRCAT model. The phylogenetic tree was visualized with GraPhlAn v. 1.1.3^95^, using the genome of *Fusobacterium hwasookii* as an outgroup to root the tree. PanPhlAn was applied using default parameters on the same raw reads and reference genomes mentioned above. We build a custom *F. nucleatum* PanPhlAn pangenome from the 56 *F. nucleatum* reference genomes by annotating each genome with Prokka v1.12^96^ and by clustering annotated genes with Roary v. 3.8^97^. Subspecies-level clusters were obtained by performing hierarchical clustering (“average” method) on the PanPhlAn presence–absence gene family matrix and by cutting at a height of 0.45. Prior to clustering, we removed gene families that were present in less than three samples or that were present in more than all-minus-three samples or reference genomes. The principal coordinates analysis of Fig. 5b was computed on the same presence–absence matrix using the jaccard distance metric. Statistical analysis was performed with custom Python and R scripts.

### Reporting summary

Further information on research design is available in the Nature Research Reporting Summary linked to this article.

## Supplementary information

Supplementary Information

Supplementary Data 1

Supplementary Data 2

Supplementary Data 3

Supplementary Data 4

Supplementary Data 5

Supplementary Data 6

Supplementary Data 7

Reporting Summary

## Data Availability

All metagenomes have been deposited and are available at the NCBI Sequence Read Archive under accession BioProject PRJNA547717.
