# Supplementary material for: Strong oral plaque microbiome signatures for dental implant diseases identified by strain-resolution metagenomics
Source: NPJ Biofilms Microbiomes. 2020 Oct 30;6:47. doi: 10.1038/s41522-020-00155-7 (PMC7603341; doi:10.1038/s41522-020-00155-7)
Supplement: Supplementary file 1 — Supplementary Information [file 41522_2020_155_MOESM1_ESM.pdf]

Strong oral plaque microbiome signatures for dental implant diseases identified by strain-resolution metagenomics

Paolo Ghensi* [1], Paolo Manghi* [1], Moreno Zolfo [1], Federica Armanini [1], Edoardo Pasolli [1], Mattia Bolzan [1,4], Alberto Bertelle [5], Federico Dell'Acqua [5], Ester Dellasega [5], Romina Waldner [5], Francesco Tessarolo [3,6], Cristiano Tomasi [2], Nicola Segata [1,^]

1. Dept. CIBIO, University of Trento, Trento, Italy
2. Dept. of Periodontology, Institute of Odontology, Sahlgrenska Academy, University of Gothenburg, Gothenburg, Sweden
3. Dept. of Industrial Engineering, University of Trento, Trento, Italy
4. Current address: PreBiomics S.r.l., Trento, Italy
5. Private Practice, Trentino-Alto Adige, Italy
6. Healthcare Research and Innovation Program (IRCS-FBK-PAT), Bruno Kessler Foundation, Trento, Italy

* Equal contribution
^ Corresponding author: nicola.segata@unitn.it

Supplementary Material

Supplementary Figures

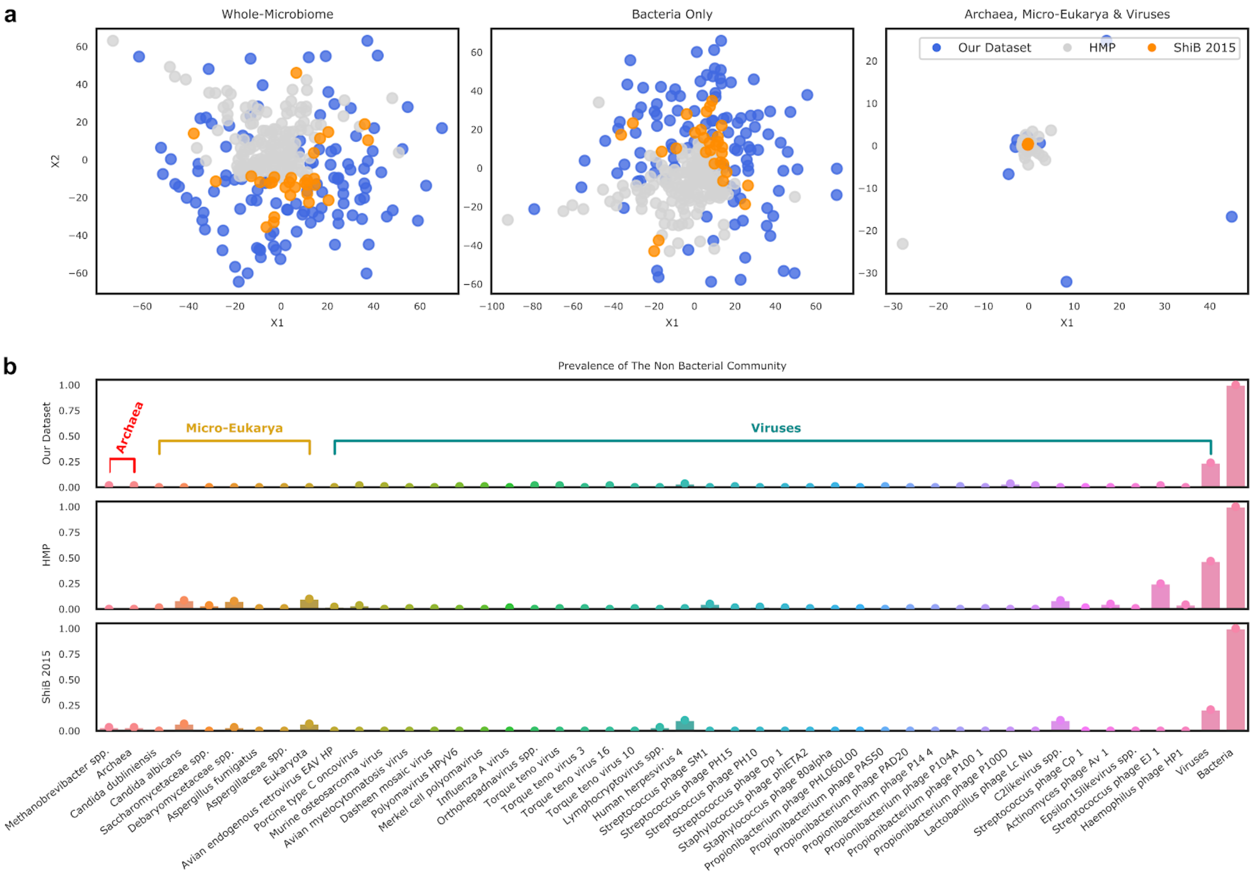

Supplementary Figure 1. Quantitative dominance of the bacterial fraction of the microbial community over archaea, micro-Eukaryotic and the viral components. a) *Ordination plot (MDS) of the microbiome, the bacteriome and the sum of all the archaea, micro-Eukarya and viruses in our dataset integrated here with the publicly available oral plaque microbiome.* ***b)*** *Distribution of the mean prevalences of the single and the taxonomically grouped taxa across datasets.*

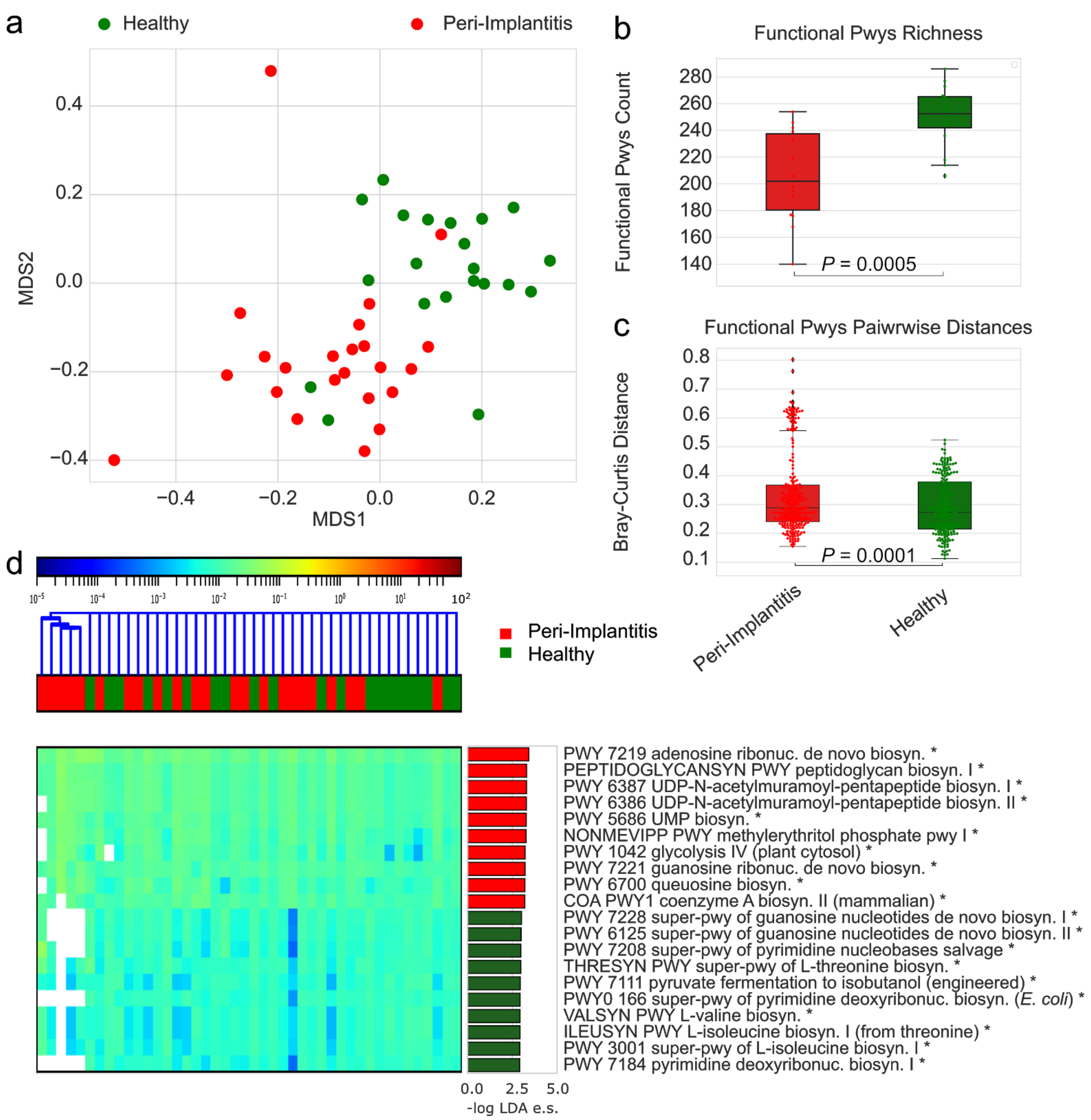

Supplementary Figure 2. Functional profiles are significantly richer but less heterogeneous among individuals in health compared to peri-implantitis. a) *Ordination plot (MDS) of healthy and peri-implantitis samples based on the presence and abundance of microbial pathways from HUMAnN2 analysis.* ***b)*** *Rarefied alpha diversities measured as the total number of pathways in each sample for the two conditions.* ***c)*** *Beta-diversities estimated with the Bray-Curtis dissimilarity metric on pathways abundance for inter-condition comparisons in the two conditions.* ***d)*** *Relative abundances and effect sizes for the 10 pathways that are the most strongly associated with either the healthy sites or the sites with peri-implantitis (top 10 species/gene families effect sizes per class).*

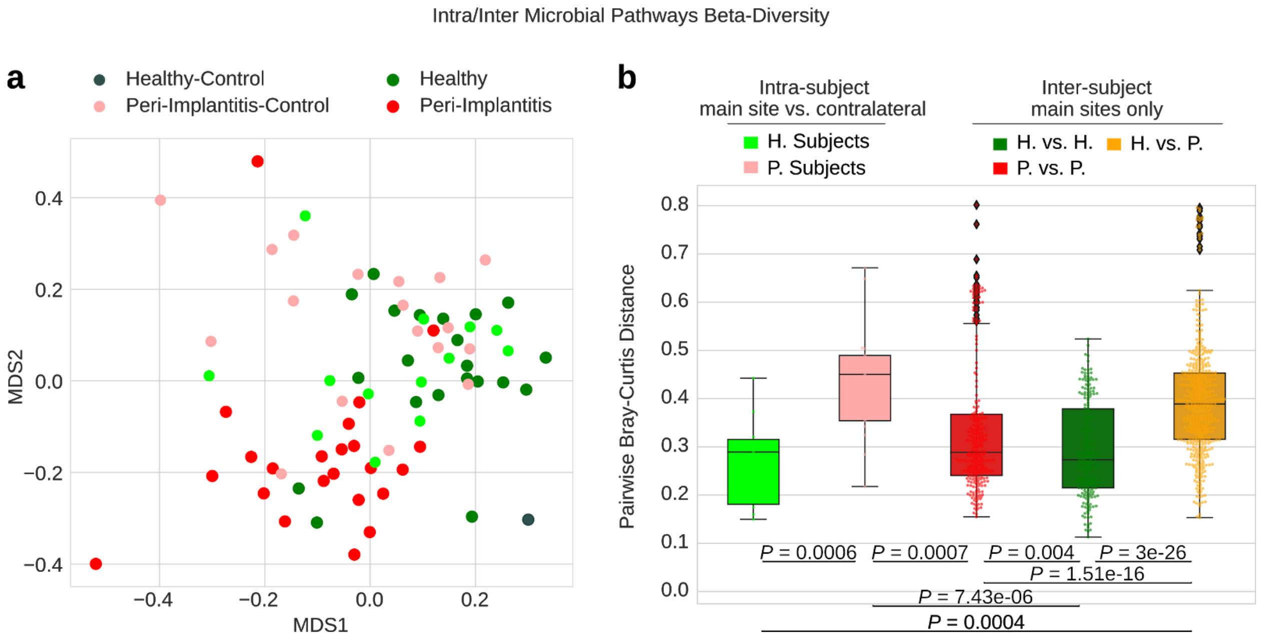

Supplementary Figure 3. The strong microbiome signature in peri-implantitis is also confirmed by the pathway analysis. a) *Ordination plot (MDS) of peri-implantitis and all healthy samples (main and contralateral) based on pathways.* ***b)*** *Intra and inter-subject beta diversity analysis based on pathways. Estimations of the intra-subject beta-diversity is performed using the main site and the contralateral samples.*

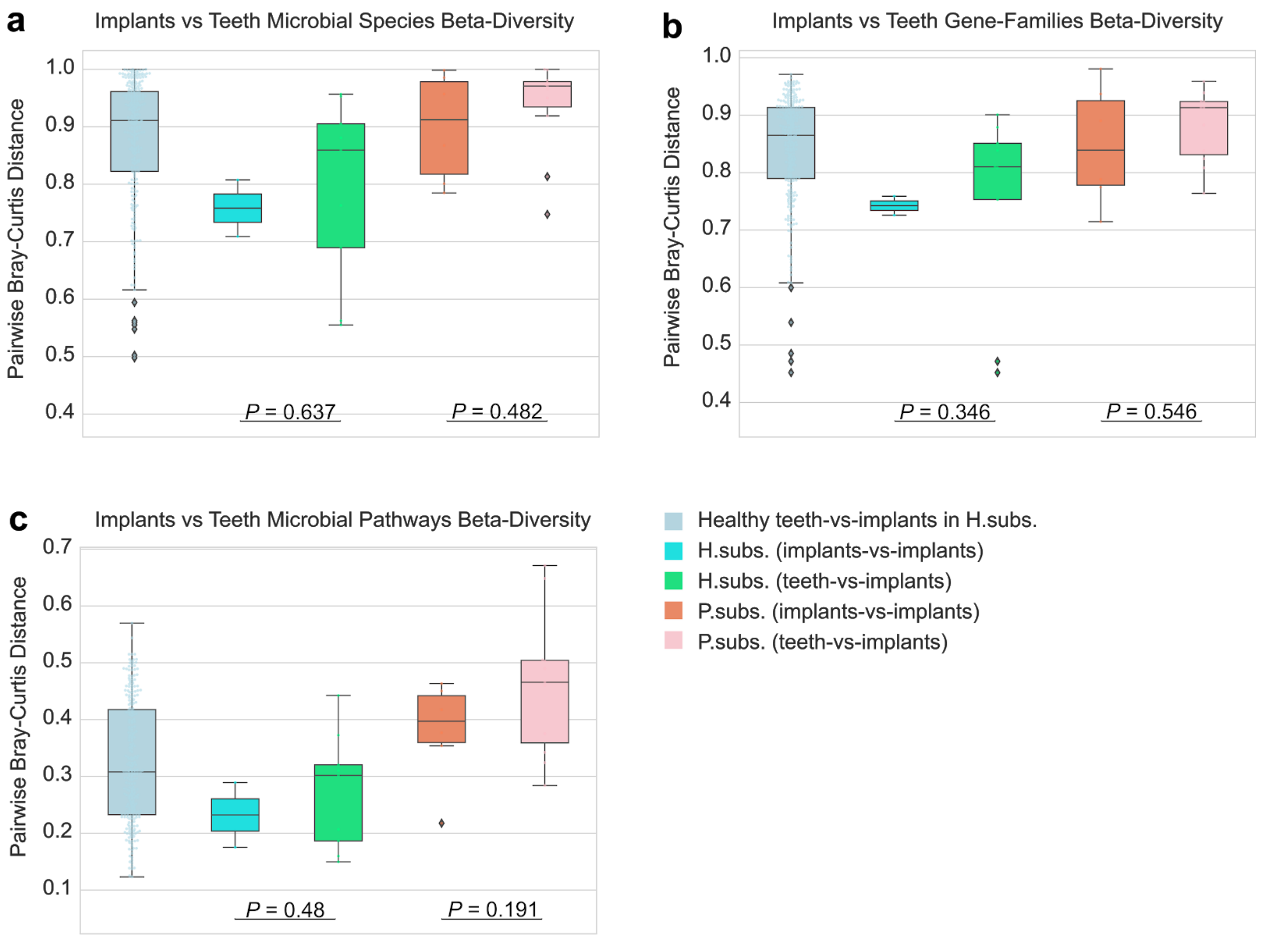

Supplementary Figure 4. Microbiome composition and functional potentials of healthy implants and teeth are not distinguishable. a) *Beta-diversity analysis for implants-teeth sample pairs based on MetaPhlAn2 quantitative taxonomic abundance,* ***b)*** *on HUMAnN2 gene-family abundances, and* ***c)*** *on HUMAnN2 pathway abundances. Estimations of the intra-subject beta-diversity is performed using the main site and the contralateral samples.*

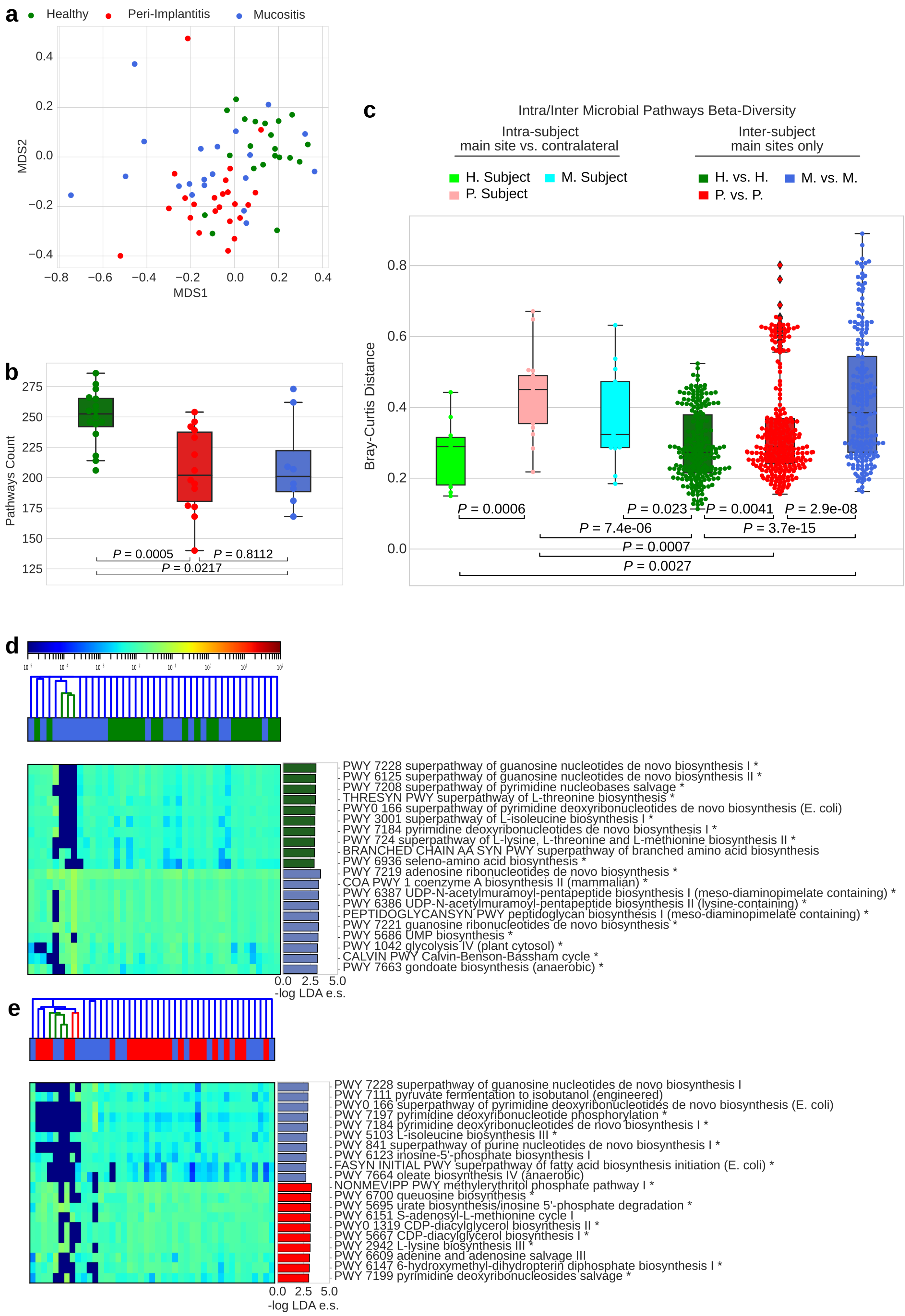
a
Healthy
Peri-Implantitis
Mucositis
MDS2
MDS1
b
Pathways Count
P = 0.0005
P = 0.8112
P = 0.0217
c
Intra/Inter Microbial Pathways Beta-Diversity
Intra-subject main site vs. contralateral
Inter-subject main sites only
H. Subject
M. Subject
P. Subject
H. vs. H.
M. vs. M.
P. vs. P.
Bray-Curtis Distance
P = 0.0006
P = 0.023
P = 0.0041
P = 2.9e-08
P = 7.4e-06
P = 3.7e-15
P = 0.0007
P = 0.0027
d
PWY 7228 superpathway of guanosine nucleotides de novo biosynthesis I *
PWY 6125 superpathway of guanosine nucleotides de novo biosynthesis II *
PWY 7208 superpathway of pyrimidine nucleobases salvage *
THRESYN PWY superpathway of L-threonine biosynthesis *
PWY0 166 superpathway of pyrimidine deoxyribonucleotides de novo biosynthesis (E. coli)
PWY 3001 superpathway of L-isoleucine biosynthesis I *
PWY 7184 pyrimidine deoxyribonucleotides de novo biosynthesis I *
PWY 724 superpathway of L-lysine, L-threonine and L-methionine biosynthesis II *
BRANCHED CHAIN AA SYN PWY superpathway of branched amino acid biosynthesis
PWY 6936 seleno-amino acid biosynthesis *
PWY 7219 adenosine ribonucleotides de novo biosynthesis *
COA PWY 1 coenzyme A biosynthesis II (mammalian) *
PWY 6387 UDP-N-acetylmuramoyl-pentapeptide biosynthesis I (meso-diaminopimelate containing) *
PWY 6386 UDP-N-acetylmuramoyl-pentapeptide biosynthesis II (lysine-containing) *
PEPTIDOGLYCANSYN PWY peptidoglycan biosynthesis I (meso-diaminopimelate containing) *
PWY 7221 guanosine ribonucleotides de novo biosynthesis *
PWY 5686 UMP biosynthesis *
PWY 1042 glycolysis IV (plant cytosol) *
CALVIN PWY Calvin-Benson-Bassham cycle *
PWY 7663 gondoate biosynthesis (anaerobic) *
-log LDA e.s.
e
PWY 7228 superpathway of guanosine nucleotides de novo biosynthesis I
PWY 7111 pyruvate fermentation to isobutanol (engineered)
PWY0 166 superpathway of pyrimidine deoxyribonucleotides de novo biosynthesis (E. coli)
PWY 7197 pyrimidine deoxyribonucleotide phosphorylation *
PWY 7184 pyrimidine deoxyribonucleotides de novo biosynthesis I *
PWY 5103 L-isoleucine biosynthesis III *
PWY 841 superpathway of purine nucleotides de novo biosynthesis I *
PWY 6123 inosine-5'-phosphate biosynthesis I
FASYN INITIAL PWY superpathway of fatty acid biosynthesis initiation (E. coli) *
PWY 7664 oleate biosynthesis IV (anaerobic)
NONMEVIPP PWY methylerythritol phosphate pathway I *
PWY 6700 queuosine biosynthesis *
PWY 5695 urate biosynthesis/inosine 5'-phosphate degradation *
PWY 6151 S-adenosyl-L-methionine cycle I
PWY0 1319 CDP-diacylglycerol biosynthesis II *
PWY 5667 CDP-diacylglycerol biosynthesis I *
PWY 2942 L-lysine biosynthesis III *
PWY 6609 adenine and adenosine salvage III
PWY 6147 6-hydroxymethyl-dihydropterin diphosphate biosynthesis I *
PWY 7199 pyrimidine deoxyribonucleosides salvage *
-log LDA e.s.

Supplementary Figure 5. Pathways analysis confirms the intermediate microbiome signature of mucositis. a) *Ordination plot (MDS) of healthy, mucositis and peri-implantitis samples based on HUMAnN2 pathway abundance.* ***b)*** *Alpha diversities measured as the total number of pathways for the three conditions.* ***c)*** *Beta-diversities estimated with the Bray-Curtis dissimilarity metric for intra- and inter-condition comparisons in the three conditions based on HUMAnN2 pathway abundance. Estimations of the intra-subject beta-diversity is performed using the main site and the contralateral samples.* ***d)*** *Relative abundances (log scale) and effect sizes (estimated using the LDA score in LEfSe) of the 10 microbial pathways most strongly discriminating healthy from mucositis sites and* ***e)*** *mucositis from peri-implantitis sites.*

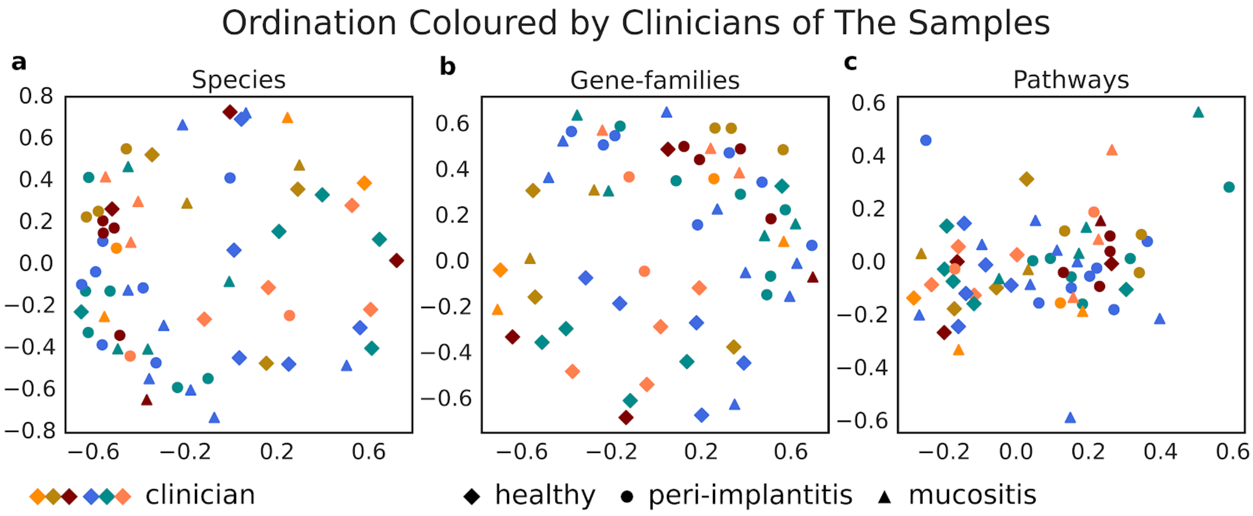

Supplementary Figure 6. Lack of strong clinician-dependent effects on the microbiome of the three main classes of this study. a) *Ordination plot (MDS) of healthy, mucositis and peri-implantitis samples based on taxonomic abundance profiles (Permanova p-value = 0.311); colours identify the different clinicians performing the sampling, shapes identify the conditions. The same analysis is performed on* ***(b)*** *microbial gene family profiles (Permanova p-value = 0.129), and* ***(c)*** *on microbial pathways' profiles (Permanova p-value = 0.623).*

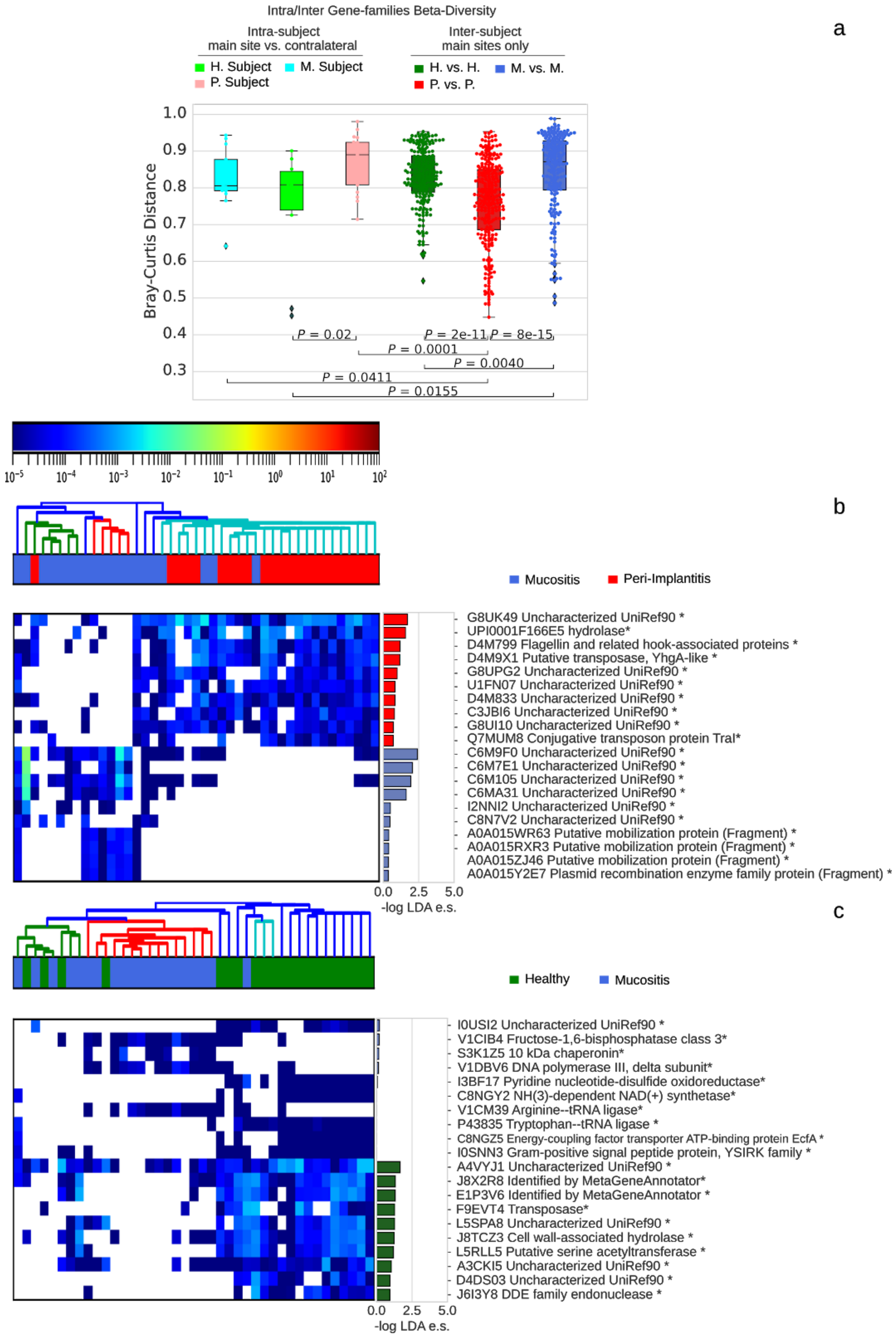
a
Intra/Inter Gene-families Beta-Diversity
Intra-subject
main site vs. contralateral
Inter-subject
main sites only
H. Subject
M. Subject
P. Subject
H. vs. H.
M. vs. M.
P. vs. P.
Bray-Curtis Distance
1.0
0.9
0.8
0.7
0.6
0.5
0.4
0.3
P = 0.02
P = 2e-11
P = 8e-15
P = 0.0001
P = 0.0040
P = 0.0411
P = 0.0155
b
Mucositis
Peri-Implantitis
G8UK49 Uncharacterized UniRef90 *
UPI0001F166E5 hydrolase*
D4M799 Flagellin and related hook-associated proteins *
D4M9X1 Putative transposase, YhgA-like *
G8UPG2 Uncharacterized UniRef90 *
U1FN07 Uncharacterized UniRef90 *
D4M833 Uncharacterized UniRef90 *
C3JBI6 Uncharacterized UniRef90 *
G8UI10 Uncharacterized UniRef90 *
Q7MUM8 Conjugative transposon protein TraI*
C6M9F0 Uncharacterized UniRef90 *
C6M7E1 Uncharacterized UniRef90 *
C6M105 Uncharacterized UniRef90 *
C6MA31 Uncharacterized UniRef90 *
I2NNI2 Uncharacterized UniRef90 *
C8N7V2 Uncharacterized UniRef90 *
A0A015WR63 Putative mobilization protein (Fragment) *
A0A015RXR3 Putative mobilization protein (Fragment) *
A0A015ZJ46 Putative mobilization protein (Fragment) *
A0A015Y2E7 Plasmid recombination enzyme family protein (Fragment) *
0.0
2.5
5.0
-log LDA e.s.
c
Healthy
Mucositis
I0USI2 Uncharacterized UniRef90 *
V1CIB4 Fructose-1,6-bisphosphatase class 3*
S3K1Z5 10 kDa chaperonin*
V1DBV6 DNA polymerase III, delta subunit*
I3BF17 Pyridine nucleotide-disulfide oxidoreductase*
C8NGY2 NH(3)-dependent NAD(+) synthetase*
V1CM39 Arginine--tRNA ligase*
P43835 Tryptophan--tRNA ligase *
C8NGZ5 Energy-coupling factor transporter ATP-binding protein EcfA *
I0SNN3 Gram-positive signal peptide protein, YSIRK family *
A4VYJ1 Uncharacterized UniRef90 *
J8X2R8 Identified by MetaGeneAnnotator*
E1P3V6 Identified by MetaGeneAnnotator *
F9EVT4 Transposase*
L5SPA8 Uncharacterized UniRef90 *
J8TCZ3 Cell wall-associated hydrolase *
L5RLL5 Putative serine acetyltransferase *
A3CKI5 Uncharacterized UniRef90 *
D4DS03 Uncharacterized UniRef90 *
J6I3Y8 DDE family endonuclease *
0.0
2.5
5.0
-log LDA e.s.

Supplementary Figure 7. Differential abundance analysis on microbial gene families shows a stronger similarity between mucositis and healthy sites, than between mucositis and peri-implantitis sites. a) *Beta-diversity distributions estimated with the Bray-Curtis dissimilarity metric for intra- and inter-condition comparisons in the three conditions.* Estimations of the intra-subject beta-diversity is performed using the main site and the contralateral samples. ***b)*** *Relative abundances (log scale) and effect sizes (estimated using the LDA score in LEfSe) of the 10 UniRef90 gene-families most strongly discriminating healthy from mucositis sites and* ***c)*** *mucositis from peri-implantitis sites.*

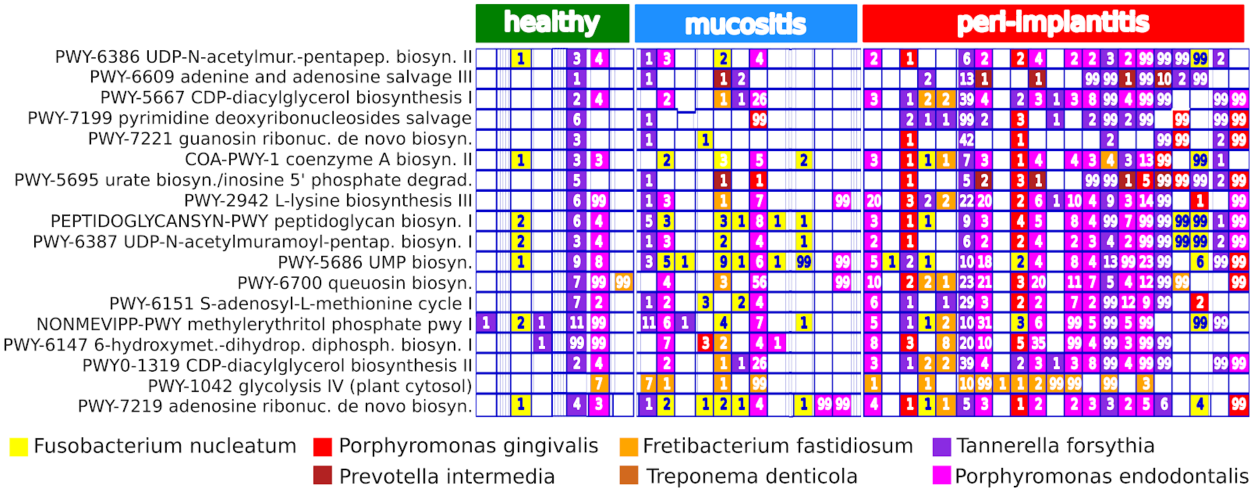

Supplementary Figure 8. Differential abundance analysis on microbial gene families shows a stronger similarity between mucositis and healthy sites, than between mucositis and peri-implantitis sites. *We report the fold-change between the relative contribution of the set of bacteria in and outside the PiRC to the 10 most differentially abundant ranking pathways for peri-implantitis versus healthy samples and versus mucositis samples. Numbers indicate the unit of the fold-change when it is higher than one. Colors indicate the member of the PiRC which is the major contributor to the pathway. We report an upper bound of 99 on the fold changes.*

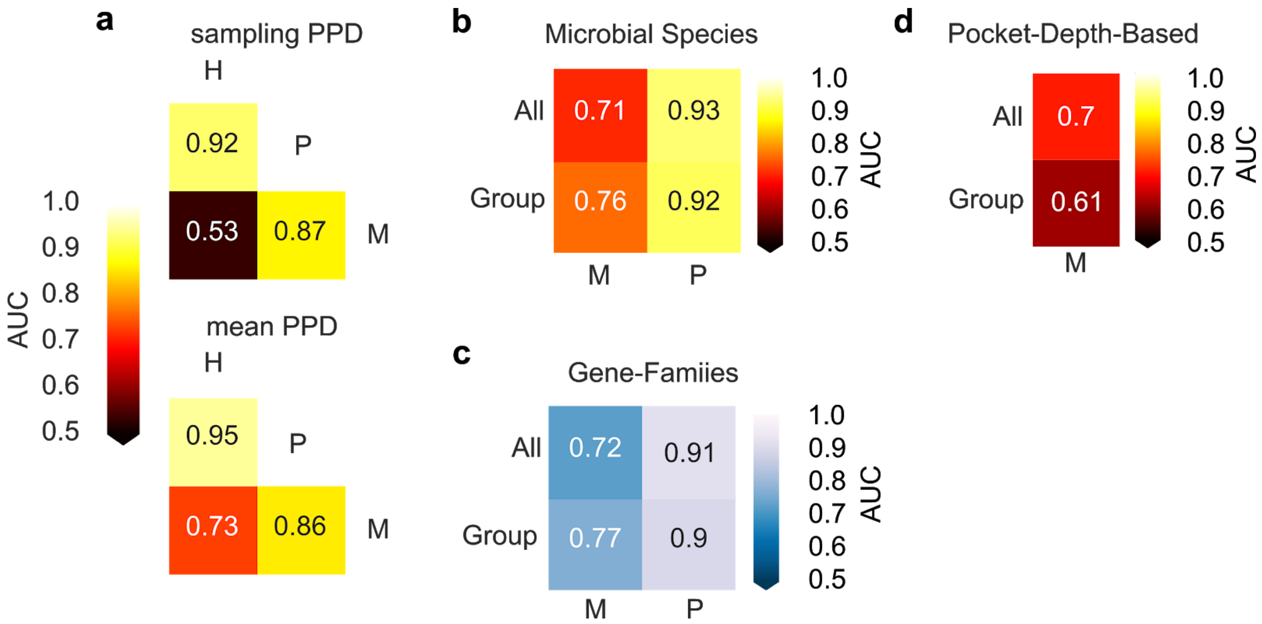

Supplementary Figure 9. Performances of peri-implant probing depth (PPD) and microbial features in predicting disease conditions. a) *Sampling-site PPD and mean-PPD used as features in a one-tree random forest for the prediction of peri-implantitis against healthy sites, mucositis against peri-implantitis, and mucositis against healthy implants.* ***b)*** *Microbial species random forests prediction of mucositis and peri-implantitis implants against the overall contralateral samples (all) and against the corresponding set of contralateral (group).* ***c)*** *Gene-families random forests prediction of mucositis and peri-implantitis implants against the overall contralateral samples (all) and against the corresponding set of contralateral (group).* ***d)*** *Mean PPD-based one-tree random forest prediction of mucositis against the entire set of contralateral samples (all) and the mucositis-group contralateral samples alone (group).*

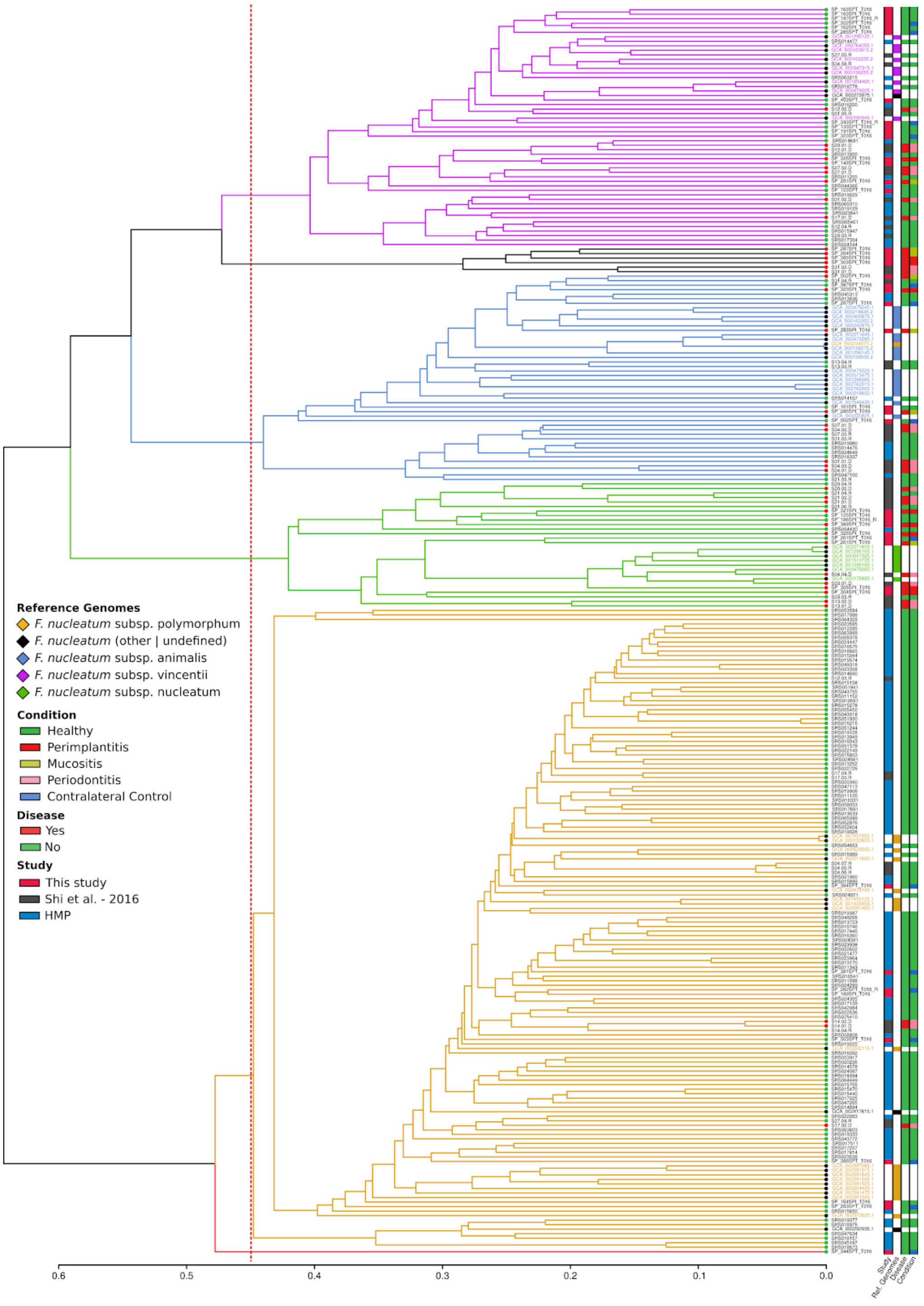

Supplementary Figure 10. Hierarchical clustering of samples based on the genomic content of different F. nucleatum subspecies. *The hierarchical clustering is based on the presence-absence matrix of gene families on 208 metagenomes. Reference genomes are added and colored by subspecies. The associated clinical metadata are presented as color-bars on the right. Six clusters were derived by cutting at a height of 0.45 to recapitulate the division of the different F. nucleatum subspecies. Gene families present in less than three or more than all-minus-three samples were excluded. Leaves colors represent the disease status of each metagenomic sample.*

Supplementary Tables

Supplementary Table S1. Experimental implants' clinical and microbial characteristics
Supplementary Table S2. Sampled teeth/implants characteristics
Supplementary Table S3. Per-sample cohort metadata with NCBI accessions
Supplementary Table S4. Statistical significance, FDR-correction, abundances and prevalences in the peri-implantitis/healthy implants comparison of microbial species
Supplementary Table S5. Lefse microbial species biomarkers for the peri-implantitis/healthy implants comparison
Supplementary Table S6. Statistical significance, FDR-correction, abundances and prevalences in the peri-implantitis/healthy implants comparison of UniRef90 gene-families
Supplementary Table S7. Lefse gene-families biomarkers for the peri-implantitis/healthy implants comparison
