# Supplementary material for: Strong oral plaque microbiome signatures for dental implant diseases identified by strain-resolution metagenomics
Source: NPJ Biofilms Microbiomes. 2020 Oct 30;6:47. doi: 10.1038/s41522-020-00155-7 (PMC7603341; doi:10.1038/s41522-020-00155-7)
Supplement: Supplementary file 9 — Reporting Summary [file 41522_2020_155_MOESM9_ESM.pdf]

nature research

Corresponding author(s): Nicola Segata

Last updated by author(s): Jul 21, 2020

Reporting Summary

Nature Research wishes to improve the reproducibility of the work that we publish. This form provides structure for consistency and transparency in reporting. For further information on Nature Research policies, see our Editorial Policies and the Editorial Policy Checklist.

Statistics

For all statistical analyses, confirm that the following items are present in the figure legend, table legend, main text, or Methods section.

n/a	Confirmed	
☐	☒	The exact sample size (n) for each experimental group/condition, given as a discrete number and unit of measurement
☐	☒	A statement on whether measurements were taken from distinct samples or whether the same sample was measured repeatedly
☐	☒	The statistical test(s) used AND whether they are one- or two-sided<br>*Only common tests should be described solely by name; describe more complex techniques in the Methods section.*
☐	☒	A description of all covariates tested
☐	☒	A description of any assumptions or corrections, such as tests of normality and adjustment for multiple comparisons
☐	☒	A full description of the statistical parameters including central tendency (e.g. means) or other basic estimates (e.g. regression coefficient) AND variation (e.g. standard deviation) or associated estimates of uncertainty (e.g. confidence intervals)
☐	☒	For null hypothesis testing, the test statistic (e.g. F, t, r) with confidence intervals, effect sizes, degrees of freedom and P value noted<br>*Give P values as exact values whenever suitable.*
☒	☐	For Bayesian analysis, information on the choice of priors and Markov chain Monte Carlo settings
☒	☐	For hierarchical and complex designs, identification of the appropriate level for tests and full reporting of outcomes
☐	☒	Estimates of effect sizes (e.g. Cohen's d, Pearson's r), indicating how they were calculated

Our web collection on statistics for biologists contains articles on many of the points above.

Software and code

Policy information about availability of computer code

Data collection	No software was used for the data collection besides the standard Illumina HiSeq 2000 operating system.
Data analysis	HUMANn ver 2.0, MetaPhLAn2.6, Strainphlan ver. 0.1 PanPhlan, ver 1.2.2 and Lefse ver 1.0, Bowtie2 v. 2.3.4, RAxML v.8.1.15, Muscle v3.8.425, Prokka v1.12, python was version 3.6.Custom python scripts used Scikit-Learn ver. 0.20.3, seaborn ver 0.9.0, Statsmodel, ver. 0.9.0., Scikit-bio python library, ver. 0.2.3, Scipy, version 1.2.1. , MetAML ver 1.1

For manuscripts utilizing custom algorithms or software that are central to the research but not yet described in published literature, software must be made available to editors and reviewers. We strongly encourage code deposition in a community repository (e.g. GitHub). See the Nature Research guidelines for submitting code & software for further information.

Data

Policy information about availability of data

All manuscripts must include a data availability statement. This statement should provide the following information, where applicable:

- Accession codes, unique identifiers, or web links for publicly available datasets
- A list of figures that have associated raw data
- A description of any restrictions on data availability

All metagenomes have been deposited and are available at the NCBI Sequence Read Archive under accession BioProject PRJNA547717).

Field-specific reporting

Please select the one below that is the best fit for your research. If you are not sure, read the appropriate sections before making your selection.

☒ Life sciences ☐ Behavioural & social sciences ☐ Ecological, evolutionary & environmental sciences

For a reference copy of the document with all sections, see nature.com/documents/nr-reporting-summary-flat.pdf

Life sciences study design

All studies must disclose on these points even when the disclosure is negative.

Sample size

Samples N. of each of the three groups in the study have been hypothesized "a priori", and dentists have been asked to collect samples until each group reached 24 units, a number sufficient to garantee statistical significance. The clinicians were also instructed to collect contralateral samples. After the discovery of the failure of a number of samples following the sampling, other main sites have been sampled in order to reach the prefixed number. The correctness of the sample group design have been assessed via statistical p-values of the several clinical variables, as clearly stated in the paper.

Data exclusions

Data that were already sequenced, preprocessed and taxonomically profiled have been excluded if they met the following conditions: they were below 50.000 reads or they were evaluated as being the result of a contamination directly looking at their metagenomic profile, by two people working in the metagenomic field, the corresponding author and the second author.

Replication

Taking advantage of a statistically adeguate number of samples, statistically significant differences between groups have been presented only if significant (p-value<0.05). Multiple testing (e.g. association of set of species and a particular condition have been FDR corrected and considered significant under a q-value of 0.05). Such statistical thresholds ensure the statistical reproducibility of the study. The machine learning have been carried out through 10 (or 5, in which case it is declared) fold cross validations.

Randomization

Each of the randomizing of the groups during the cross-validations has been repeated 20 times. This procedure ensures an adequate averaging over the final result.

Blinding

The investigators carrying out the study were not the same people having collected the samples. The clinicians who collected the samples wer not responsible of the amount of sample in each class of disease they were collecting, as this was prerogative of the investigators carrying out the study. The absence of statistical bias due to differences among the clinicians has been established visually and statistically with a supplementary figure.

Reporting for specific materials, systems and methods

We require information from authors about some types of materials, experimental systems and methods used in many studies. Here, indicate whether each material, system or method listed is relevant to your study. If you are not sure if a list item applies to your research, read the appropriate section before selecting a response.

Materials & experimental systems

n/a	Involved in the study	
☒	☐	Antibodies
☒	☐	Eukaryotic cell lines
☒	☐	Palaeontology and archaeology
☒	☐	Animals and other organisms
☐	☒	Human research participants
☒	☐	Clinical data
☒	☐	Dual use research of concern

Methods

n/a	Involved in the study	
☒	☐	ChIP-seq
☒	☐	Flow cytometry
☒	☐	MRI-based neuroimaging

Human research participants

Policy information about studies involving human research participants

Population characteristics

As written in the methods: "Inclusion criteria involved good general health as evidenced by themedical history, being at least 18 years of age, not fewer than 8 teeth, at least one functioningoral implant restored with crowns or prostheses for at least 1 yr, willingness to participate in thestudy. Exclusion criteria included pregnancy or lactation, HIV, use of immunosuppressantmedications, bisphosphonates, or steroids, use of chlorhexidine mouthwash or gel during theprevious 2 weeks, oral prophylactic procedures within the preceding 3 months, intake ofsystemic antibiotics or probiotics within the past 6 months."

Recruitment

As stated in the methods: "Patients were identified and selected in the different private practices and were included in oneof the following study groups according to the state of health of their dental implants: a) healthy(H, patients with at least one healthy implant and no implants with mucositis or peri-implantitis),b) mucositis (M, patients with at least one implant with mucositis and no implants withperi-implantitis), c) peri-implantitis (P, patients with at least one implant with peri-implantitis).

Theselection and inclusion of patients in one of the groups was based on radiographic evaluation ofthe marginal bone level, clinical signs of inflammation, and/or presence of suppuration accordingto the criteria delineated by the Consensus Report on Peri-implant Diseases"

Ethics oversight	This study was approved by the ethics committee of the University of Trento (no. 2015-024) andwas conducted in accordance with the guidelines of the World Medical Association Declarationof Helsinki.

Note that full information on the approval of the study protocol must also be provided in the manuscript.
